# Comparative metabolomic profiling in the roots and leaves in contrasting genotypes reveals complex mechanisms involved in post-anthesis drought tolerance in wheat

**DOI:** 10.1371/journal.pone.0213502

**Published:** 2019-03-11

**Authors:** Zhiyu Kang, Md Ali Babar, Naeem Khan, Jia Guo, Jahangir Khan, Shafiqul Islam, Sumit Shrestha, Dipendra Shahi

**Affiliations:** 1 College of Agronomy and Biotechnology, Yunnan Agricultural University, Kunming, Yunnan Province, China; 2 Agronomy Dept., University of Florida, Gainesville, FL, United States of America; 3 Plant Science, Quaid-i-Azam University, Islamabad, Pakistan; Louisiana State University, UNITED STATES

## Abstract

Understanding the contrasting biochemical changes in different plant parts in response to drought can help to formulate smart strategies to develop drought tolerant genotypes. The current study used metabolomics and physiological approaches to understand the differential biochemical changes coupled with physiological adjustments in leaves and roots to cope with drought stress in two wheat genotypes, LA754 (drought tolerant) and AGS2038 (drought sensitive). The gas chromatography-mass spectrometry (GC-MS) analysis and physiological trait estimation were performed in the roots and leaves after drought imposition. Drought induced reduction was observed in all physiological and yield related traits. In LA754, higher numbers of metabolites were altered in leaves (45) compared to roots (20) which indicates that plants allocated more resources to leaves in tolerant genotype. In addition, the metabolic components of the root were less affected by the stress which supports the idea that the roots are more drought tolerant than the leaf or shoot. In AGS2038, thirty and twenty eight metabolites were altered in the leaves and roots, respectively. This indicates that the sensitive genotype compromised resource allocation to leaves, rather allocated more towards roots. Tryptophan, valine, citric acid, fumaric acid, and malic acid showed higher accumulation in leaf in LA754, but decreased in the root, while glyceric acid was highly accumulated in the root, but not in the leaf. The results demonstrated that the roots and shoots have a different metabolic composition, and shoot metabolome is more variable than the root metabolome. Though the present study demonstrated that the metabolic response of shoots to drought contrasts with that of roots, some growth metabolites (protein, sugar, etc) showed a mirror increase in both parts. Protein synthesis and energy cycle was active in both organs, and the organs were metabolically activated to enhance water uptake and maintain growth to mitigate the effect of drought.

## Introduction

Among the abiotic stresses, drought is the most important environmental stress that limits growth, distribution, and yield, and has become a serious problem in global food security [1; 2; 3]. Moreover, the global climate change including high temperature and unpredictable rainfall pattern coupled with the increasing world human population is creating immense pressure on food security and sustainability [2]. To cope against these challenging situations, breeding for more drought resilient wheat has been identified as a major sustainable approach [2]. The potential yield of the crop plants may decrease more than 50% due to combine effect of drought with other abiotic stresses [4]. From the simulated crop modeling, it is predicted that by the end of the twenty-first century, the drought affected areas under cultivation may be doubled than present [5; 6].

Drought during the flowering stage and onwards up to grainfilling in wheat typically accelerates senesces and reduces grain yield and number by interrupting photosynthesis and by increasing translocation of carbohydrates [7]. Drought differentially affects different plant parts, such as root and shoot. The above ground parts (shoots) are autotropic and below ground (roots) plants are heterotrophic in nature and have different functions. Roots act as anchor for the plants, and play a major role for water and nutrient uptake [8]. Contrary to that, shoots produce assimilates through photosynthesis where plants capture carbon and light to produce carbohydrate [8]. The leaves and roots of the plants respond differently to the abiotic stresses [9; 10; 11]. At shoot level, drought affects morphological, chemicals, and physiological traits including stomata, water-use efficiency; relative water content, evapotranspiration efficiency, abscisic acid levels, cell membrane stability, and carbon isotope discrimination. While at root level, drought reduces root length, density, and dry weight that reduces water uptake capacity and causes stomatal closure to reduce water loss from the plants that eventually leads to lower photosynthetic rate by diminishing internal CO2 concentration [12; 13; 14]. Under irrigated and high nutrient available conditions, plants distribute more resources towards shoot than roots [15], while under drought condition, more resources allocation towards root has been observed by different authors [16; 17]. Higher root growth also has been observed under reduced nitrogen supply as well [18]. It is extremely important to determine the mechanisms by which plants improve tolerance against stress, particularly the complex and intricate regulatory mechanisms that coordinate the demands of physiological activity, growth, and development between the above and below ground plant parts [19].

The genetic improvement can be easy to understand with the biochemical processes controlling those traits that are associated with the drought tolerance mechanism [20]. Under abiotic stress, plants produce an array of biomolecules including different metabolites. Plants also modify their physiological mechanisms under abiotic stress to adapt to the changed environmental condition through metabolic homeostasis. Plant metabolites play an essential role in plant growth and development under stress through cell integrity, energy storage, cell signaling, membrane formation and scaffolding, and through whole-plant resource allocation [21]. When comparing synthesis or allocation of different metabolites between root and shoot in 2 grass species under contrasting water regimes, it was evident that the different primary metabolites like sugars, amino acids, and fatty acids were synthesized mainly in photosynthetic tissues under irrigated condition, but concentration of these metabolites were low in root [22]. Contrary to that, under drought stress, synthesis or allocation of several primary metabolites were increased at roots, whereas it decreased in shoots. In another comparative metabolic profiling study in root and shoot on 7 *Triticum* species, Ullah et al. [23] noticed altered sugars, amino acids, organic acids, and low molecular weight compounds levels in both leaf and root samples of different Triticeae species under drought stress. A recent study in the leaf, stem, root collar, and root under drought stress in drought tolerant plant *Caragana korshinskii*, several hundred metabolites were identified with differing levels depending on the organs [24]. Their study demonstrated that stress induced an increased level of various small carbohydrates and soluble amino acids in each of the 4 organs (leaf, stem, root collar, and root). Different amino acids as well as intermediates of Krebs cycle and glycolysis showed a reduction at the whole plant level due to drought stress. Their study revealed that key metabolic (such as energy metabolism) and physiological (such as photosynthesis) mechanisms were compromised due to drought stress and each organ employed a distinct strategy to cope with drought stress.

Though there is clear evidence of allocation or synthesis of different metabolites in different plant parts under stress environments, but there is very little information on how these allocations and syntheses differ at the genotypic level. Though post-anthesis drought stress contributes significant damage to wheat productivity, but there is little information available on comparing differential metabolic alterations in root and leaf in different genotypes for post- anthesis draught stress tolerance. The main objectives of the present study were to compare genetic variations in metabolic changes in leaves and roots in drought sensitive and tolerant wheat genotypes those subjected to post-anthesis drought stress.

## Materials and methods

### Genetic material and growth conditions

Two US wheat genotypes, LA754 (drought tolerant) and AGS2038 (drought sensitive), were used for the study. These genotypes are commercially grown in the southeastern United States and have been developed by Louisiana State University, located in Baton Rouge, Louisiana (LA754) and the University of Georgia in Athens, Georgia (AGS2038). Both are early maturing (112–115 Julian days), medium height (86–91 cm), and high yielding (5800–6200 kg ha^-1^) wheat varieties. In the field and greenhouse-based evaluation, LA754 demonstrated better drought tolerance than AGS2038 with minimum damage to photosystem, chlorophyll content, and cell membrane stability. Seeds of genotypes were collected from University of Georgia and Louisiana State University small grain breeding programs and were vernalized at 4°C for 4 weeks prior to planting to induce flowering. Each genotype was planted in 20 pots (30 cm diameter and 28 cm height; filled with Metro-Mix, Sun Gro Horticulture, Agawam, MA, USA, artificial media with) with 3 plants per pot and was grown in a controlled greenhouse condition located at the University of Florida, in Gainesville, Florida. The growing conditions were maintained at day/night (16 h/8 h) temperature of 20/15 ± 0.5°C and relative humidity of 60 ± 2%. The pots were watered at 3-day intervals until imposition of drought stress, and fertilized with 2 splits of 12 g of Scott’s Osmocote (20N-4P-8K) during the study period. Drought stress was imposed at 7 days after anthesis, when most tillers were flowering, by withdrawing watering. Pots were randomly assigned into 2 different groups (10 pots/group): drought stress and control (well-watered). The experiment was laid out in a completely randomized design where each genotype contained 5 pots/treatment and was randomly assigned within each treatment for two genotypes.

### Estimation of physiological traits

#### Chlorophyll fluorescence (Fv/Fm), chlorophyll content, and membrane thermostability

Chlorophyll fluorescence (the ratio of variable fluorescence, Fv, to maximum fluorescence Fm) and chlorophyll content was measured on intact flag leaves of both control and drought treated plants at 14 and 21 days after drought imposition to assess the thylakoid membrane and chlorophyll damages [25; 26]. The lower Fv/Fm ratio indicates higher damage to photosystem II due to stress at the thylakoid membrane. Chlorophyll fluorescence was measured at one-third the length from the base on the abaxial surface using a pulse modular chlorophyll fluorometer (Model OS30P, Opti-Sciences, Hudson, New Hampshire, USA) after 30 minutes of dark adaptation. The current study compared the Fv/Fm values between control and drought stressed plants to assess damage to thylakoid membrane and the relative damage was estimated as [(Fv/Fm _control_—Fv/Fm _drought_)/ Fv/Fm _control_]*100 [26]. The chlorophyll content was measured at the same area of the same flag leaves used for fluorescence measurements. The relative damage to chlorophyll content was estimated by comparing SPAD values between control and drought treated plants and expressed as [(SPAD _control_-SPAD _drought_)/SPAD _control_]*100 [26]Membrane thermostability was assessed using the method described by Ristic and Cass [27]. Six leaf disks (diameter = 5 mm) were collected from flag leaves for each biological replication at 14 and 21 days after drought treatment [27]. Then the leaf disks were put in de-ionized water (25 mL) in sealed vials and shaken on a linear shaker at 5°C for 20 hours. The Accumet research AR50 conductivity meter (Fisher Scientific) was used to measure the electrical conductivity (μS/cm) of the aqueous solution after standardization with 1M CaCl2. The leaf tissues were then autoclaved for 20 min (120°C, 15 psi) and then shaken for 20 hours at 5°C. The conductivity of the solution was again measured and the percent electrolyte leakage was calculated based on the conductivity before and after autoclaving. The membrane thermostability was calculated as 100 × (%Ld -%Lc)/(X-%Lc), where d was drought stressed, c was control, and ‘X’ was the percent leached value corresponding to 100% damage that was assumed to be 100% leached [27].

The chlorophyll fluorescence, chlorophyll content, membrane thermostability were measured at 3 flag leaves (3 different plants/pot) and average value of three readings was considered as a single biological replication for further statistical analysis. Five replicated values/genotype were used for statistical analysis and comparison of treatment means, and significant testing at *P* < .05 level [28].

### Morphological traits

#### Measurements of growth and yield

Shoots from a single plant were collected, cutting at the base for each biological replication under both control and drought treatments at 14 and 21 days after stress imposition. Then the samples were oven-dried at 50°C for 7 days and weighed, and shoot dry per tiller was estimated by dividing total dry weight by the number of tillers [29]. The roots of the same plants with soil were isolated from the pots, washed carefully to separate roots, dried at 50°C for 7 days [28]. The root dry weights were measured after 21 days of drought stress imposition. The shoot and root dry weight was estimated for five replications per genotype per treatment. When plants reached physiological maturity, they were harvested for each replication under both treatments and oven- dried at 50°C for 7 days. The threshing was performed manually to minimize seed loss. Grain weight per spike was estimated by dividing total grain weight/plant by number of spikes/plant. The harvest index (HI) was calculated dividing grain weight by total biomass. The number of grains/spike was calculated by dividing total grains/plant by number of spike. A random 200 grains were counted and weighed to estimate 1000 grain weight. The drought induced reduction to yield and growth related traits was calculated by comparing values between control and drought treated plants and expressed in percent as follows: percent reduction = [((control values)-(drought-stressed values))/(control values)] *100 [29].

#### Sample preparation for metabolomic analysis by GC-MS

Flag leaves and roots of both genotypes were harvested for metabolomic analyses from 3 biological replications (3 pots) under control and drought treatments at 14 and 21 days after post- anthesis drought treatment. Leaf and root tissues were flash-frozen in liquid N2 immediately after collection and stored at -80 oC. Samples were lyophilized and ground using a tissue lyser (24 leaves and 24 root samples/genotype) and then stored at -80°C until processing [28].

GC/MS analysis was performed by using protocol described by South et al [30]. For derivatization prior to GC/MS analysis, the dried 80% methanol extracts were treated with 80 μL of methoxyamine hydrochloride (40 mg.mL^−1^ in pyridine) at 40°C for 60 min, followed by the addition of 120 μL N-Methyl-N-(trimethylsilyl) trifluoroacetamide (Pierce Biotech., Rockford, Illinois) for 90 min at 50°C was used for the analysis [30]. GC was performed using a HP-5MS capillary column (60 m × 0.32 mm × 0.25 μm) with an Agilent 7890 GC coupled with 5975 MSD (Palo Alto, California). The inlet and MS interface temperatures were kept at 230°C and the ion source temperature was adjusted to 230 oC. One microliter of the derivatized extract was injected with a split ratio of 20:1 into the GC column using He as a carrier gas at a constant flow rate of 2.4 mL min^−1^. The temperature program was set to an initial 5 min isothermal heating at 70°C, followed by an oven temperature increase of 5°C min^−1^ to 310°C, and finally hold for 10 min at 310°C. The mass spectrometer was operated in positive electron impact (EI) mode at 69.9 eV ionization energy in *m/z* 50–800 scan range. The spectra of all chromatogram peaks were evaluated using the Automated Mass Spectral Deconvolution and Identification System (AMDIS) (National Institute of Standards and Technology, NIST), Gaithersburg, Maryland) program. The mass spectra of all chromatogram peaks were compared with EI spectra of a custom built library. To allow comparisons between samples, all data were normalized to the internal standard (hentriacontanoic acid at 10 mg.mL^−1^ Sigma, St. Louis, Missouri) in each chromatogram, and the dry weight of each sample [30].

#### Metabolic data analysis

The relative concentrations (g/DW) of different metabolites for leaf and root tissues of each biological replication under both drought and control conditions at 14 and 21 days after drought stress treatment were formatted as comma separated values (.csv) files. The cvs file was uploaded to the MetaboAnalyst 3.0 server (http://www.metaboanalyst.ca) [31] for successive analysis. To improve data quality for performing downstream statistical analysis, the data quality was checked and normalized by sum, log transformation and auto scaling [31]. in. To maximize the differences and to detect those differences in metabolic profiling between control and drought treated group of plants, the current research applied a multivariate method, Partial Least Squares Discriminant Analysis (PLS-DA). Two different methods, Significant Analysis of Metabolites (SAM) and PLS-DA, were separately used to identify the most important metabolites associated with stress and genotypes [28]. Multifactorial ANOVA was performed to identify metabolite that altered significantly in different conditions (control and drought), or in different genotypes (tolerant and sensitive), or in 2 time points of sampling (14 and 21day after draught stress treatment). Genotype, irrigation conditions, and sampling time were considered as fixed effects. A t-test was applied to calculate fold change (FC) and false discovery rate (*P ≤ 0*.*05*) of the metabolites for leaf and root between 2 groups means (drought over control) in tolerant and sensitive genotypes [28]. A pathway analysis was performed to better elucidate the function of the altered (significantly changed at *P < 0*.*05*) metabolites by using MetaboAnalyst 3.0, via Kyoto Encyclopedia of Genes and Genomes (KEGG) pathway database (http://www.genome.ad.jp/kegg/pathway.html) compared with *Oryza sativa* ssp. *japonica* (Rice Annotation Project Data Base http://rapdb.dna.affrc.go.jp) pathway library [28]. Analysis of variance was calculated with assistance of ‘lme4’ and ‘lsmeans’ package in R [32].

## Results

### Physiological traits

During present study, drought stress caused a significant (*p* < 0.05) reduction in the chlorophyll content and membrane thermostability in both tolerant (LA754) and sensitive (AGS2038) genotypes as compared to control (Table 1), and the reduction was increased with the progression of drought stress. The photochemical efficiency of PSII (Fv/Fm) showed minimum reduction due to drought stress in tolerant genotype compare to control condition. On the contrary, the sensitive genotype showed significant reduction in Fv/Fm values due to drought stress compared to control well water condition. Though drought caused reduction to all those 3 physiological traits; however, the reduction was significantly higher in the sensitive genotype (AGS2038) (34.2 to 54.2%, 36.7 to 60.1%, and 28.4 to 50.9%, respectively, for Fv/Fm, chlorophyll content, and membrane thermostability) compared to the tolerant genotype (LA754) (4.2 to 4.9%, 10.7 to 33.1%, and 7.4 to 12.2%, respectively (Table 1).

**Table 1:** Mean (±SE) of chlorophyll florescence (Fv/Fm ratio), SPAD chlorophyll content and membrane thermostability, and percent (%) reduction due to stress in two wheat genotypes under control and drought conditions at 14 and 21 days after water stress imposition.

Traits	LA754						AGS2038					
	14-days			21-days			14-days			21-days		
	Control	Drought	Reduction (%)	Control	Drought	Reduction (%)	Control	Drought	Reduction (%)	Control	Drought	Reduction (%)
Chlorophyll florescence (Fv/Fm)	0.736±0.05	0.706±0.05	4.2	0.677±0.05	0.644±0.05	4.9	0.716±0.05	0.469±0.05	34.5	0.703±0.05	0.322±0.05	54.2
SPAD chlorophyll content (SCC)	49.1±1.7	43.8±1.7	10.7	47.8±2.8	32.0±2.8	33.1	44.7±1.7	28.3±1.7	36.7	42.3±2.8	16.5±2.8	60.1
Membrane thermostability (%)			7.4±3.1			12.2±3.8			28.4±3.1			50.9±3.8

### Growth and yield related traits

Table 2 presents the mean ± standard error (SE) for shoot dry weight/tiller, root dry weight/plant, grain weight/spike, harvest index (HI), numbers of grains/spike (NGS) and thousand-grain weight (TGW). Both genotypes showed significant reduction under drought stress compared to the control condition. Though both genotypes were significantly affected by drought stress, however, the percent reduction of growth and yield related traits were was higher in sensitive genotype AGS2038 (62.9% for shoot dry weight/tiller, 40.7% for root dry weight/plant, 63.2% for grain weight per spike, 12.3% for HI, 32.7% for NGS, and 54.4% for TGW) compared to the tolerant genotype LA754 (35.2% for shoot dry weight/tiller, 23.1% for root dry weight/plant, 15.7% for grain weight per spike, 7.3% for HI, 22.2% for NGS, and 16.3% for TGW) (Table 2).

**Table 2:** Mean (±SE) of yield related traits and percent (%) reduction due to stress in two wheat genotypes under control and drought conditions.

Traits	LA754			AGS2038		
	Control (Mean±SE)	Drought (Mean±SE)	Reduction (%)	Control (Mean±SE)	Drought (Mean±SE)	Reduction (%)
Shoot dry weight/tiller (SDWT, g)	3.4±0.48	2.2±0.48	35.2	7.0±0.3	2.6±0.3	62.9
Root dry weigh/plant (g)	12.1±0.92	9.3±0.78	23.1	13.5±1.2	8.1±0.62	40.7
Grain weight/spike (GWS, g)	1.9±0.12	1.6±0.12	15.7	1.9±0.12	0.70±0.12	63.2
Harvest index (HI, %)	54.8±1.8	50.8±1.8	7.3	29.9±1.8	26.2±1.8	12.3
Grains/spike (GNS)	28.8±1.8	22.4±1.8	22.2	46.4±1.8	31.2±1.8	32.7
1000- grain weight (TGW, g)	53.5±3.65	44.8±3.65	16.3	53.5±3.6	24.4±3.6	54.4

### Profiling of metabolites

**Fig 1:**
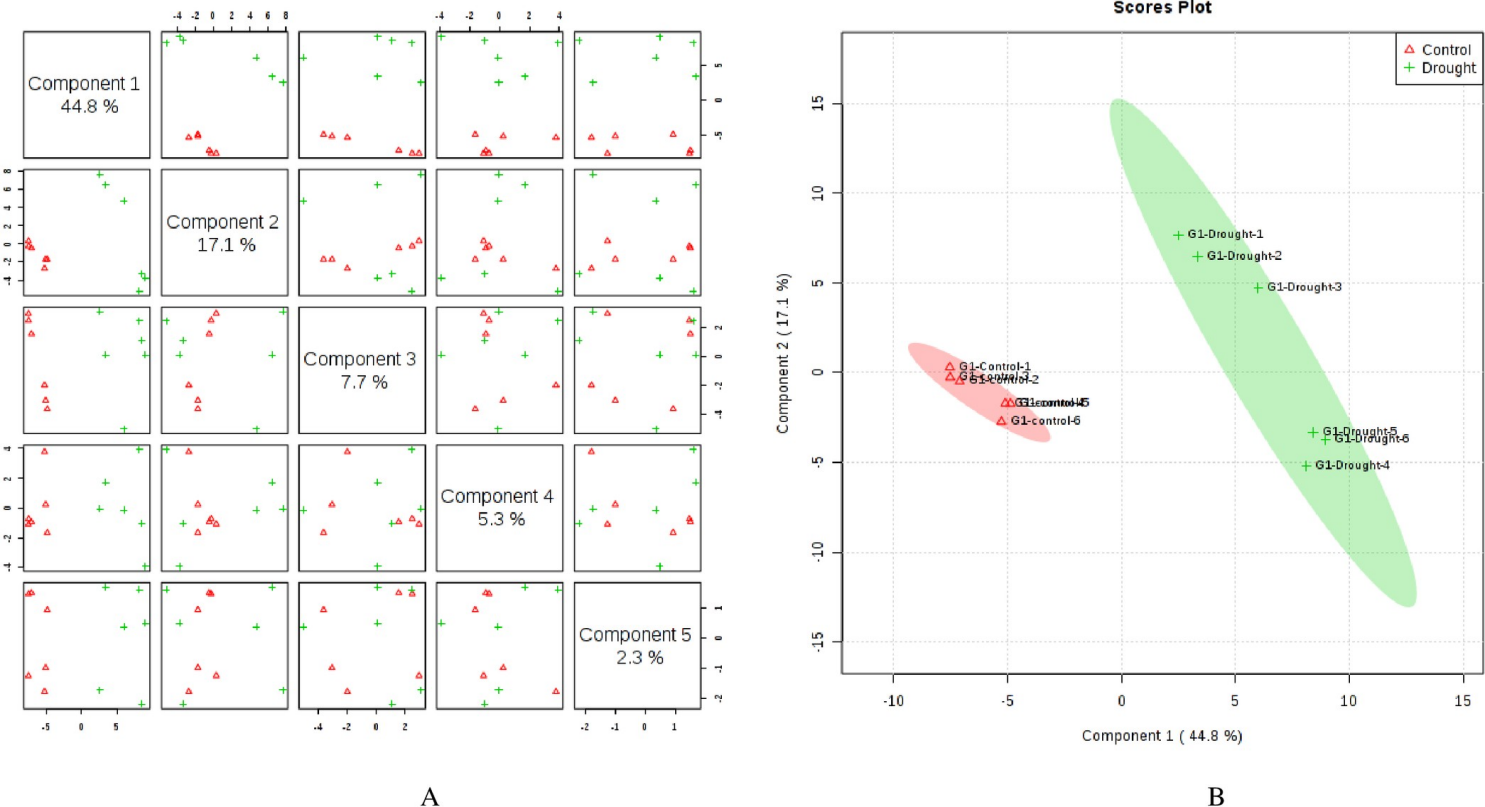
Partial least square discriminant analysis (PLS-DA) (A) and 2D Scores plot (B) in flag leaves of LA754 under control (irrigated) and drought conditions. Samples at control and drought treatments did not overlap with each other indicating an altered state of metabolite levels in the wheat leaves.

**Fig 2:**
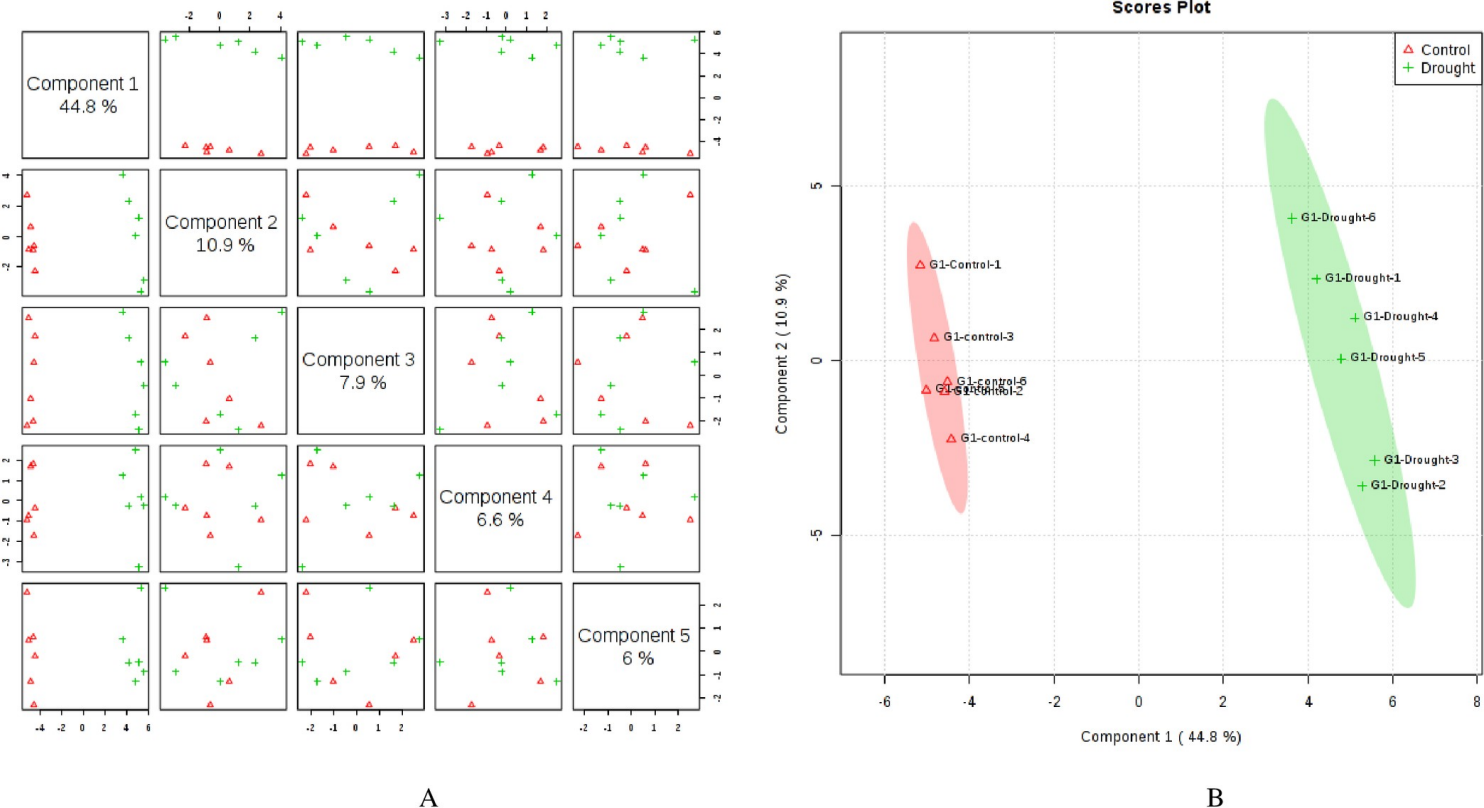
Partial least square discriminant analysis (PLS-DA) (A) and 2D Scores plot (B) in roots of LA754 under control (irrigated) and drought conditions. Samples at control and drought treatments did not overlap with each other indicating an altered state of metabolite levels in the wheat roots.

**Fig 3:**
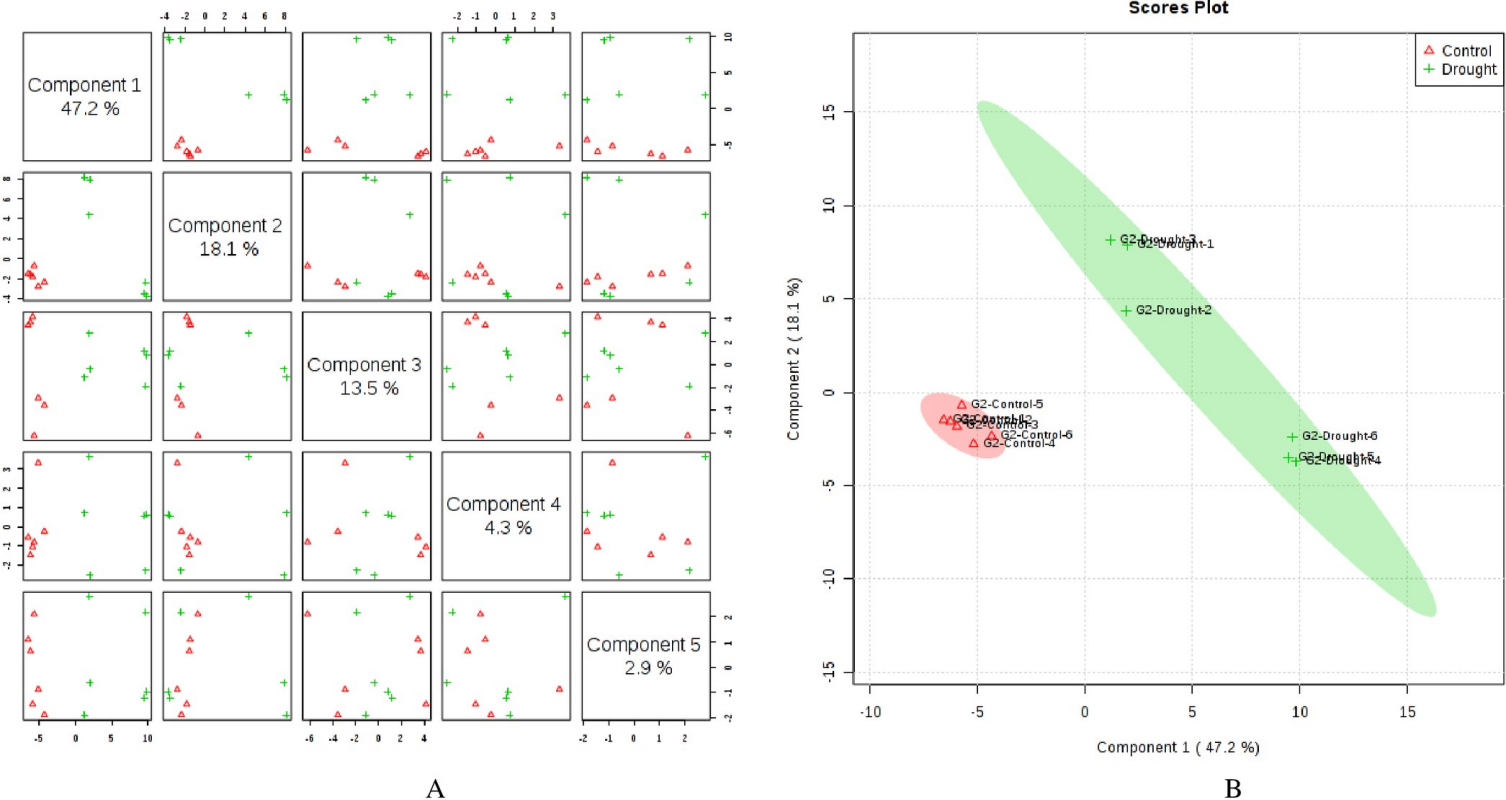
Partial least square discriminant analysis (PLS-DA) (A) and 2D Scores plot (B) in flag leaves of AGS2038 under control (irrigated) and drought conditions. Samples at control and drought treatments did not overlap with each other indicating an altered state of metabolite levels in the wheat leaves.

**Fig 4:**
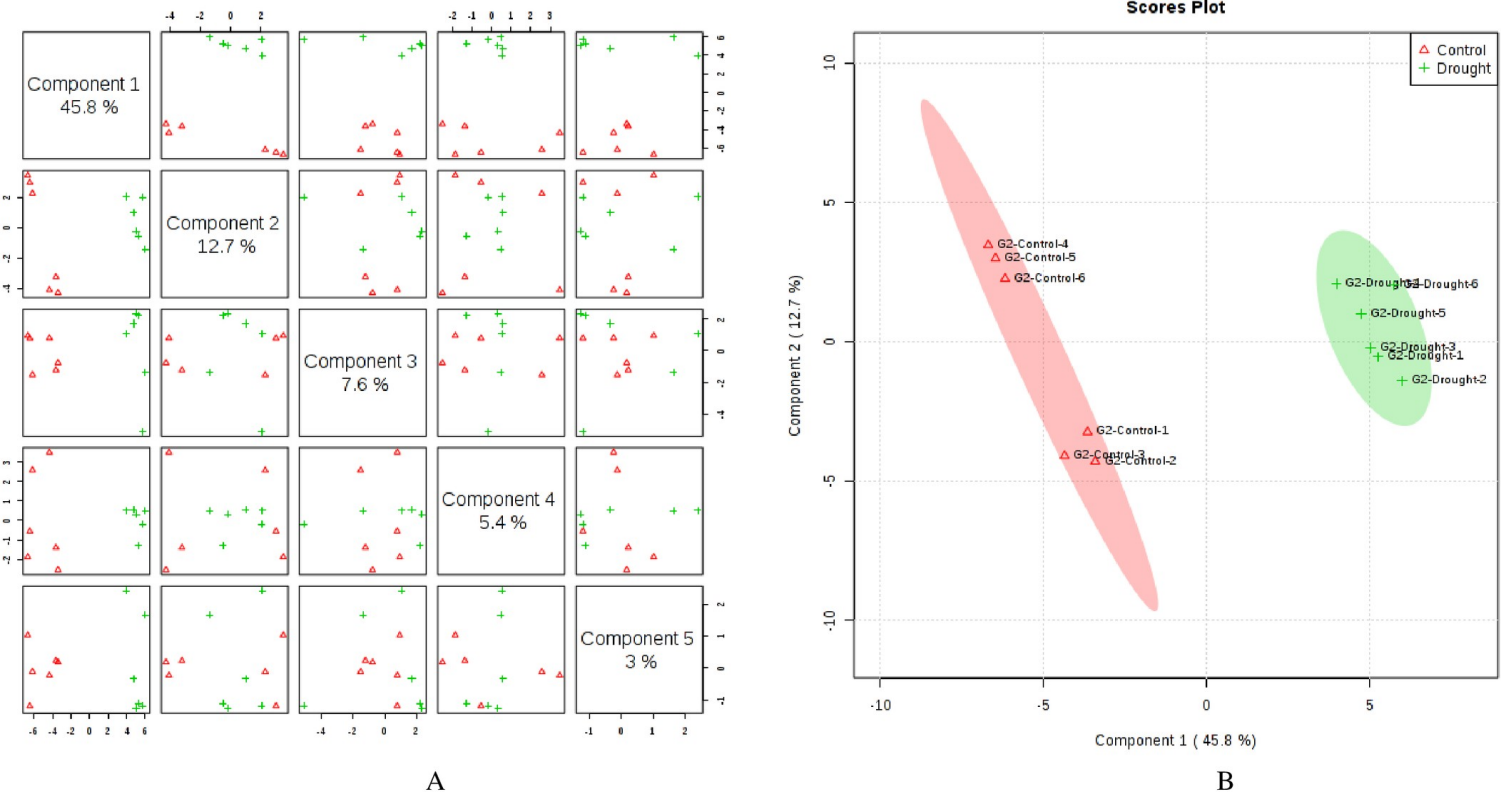
Partial least square discriminant analysis (PLS-DA) (A) and 2D Scores plot (B) in roots of AGS2038 under control (irrigated) and drought conditions. Samples at control and drought treatments did not overlap with each other indicating an altered state of metabolite levels in the wheat roots.

Metabolite profiling by GC-MS identified a total of 142 known compounds from wheat flag leaves and 99 from roots across genotypes that included amino acids, sugars, organic acids, organic compounds, fatty acids, amines, vitamins, and other compounds. Supervised clustering method analysis, Partial Least Squares Discriminant Analysis (PLS-DA), was performed for each genotype after post-anthesis drought stress in both flag leaves and roots separately. In flag leaves of LA754, 5 PLS-components (PCs) explained 77.2% of the total variation, in which the first and second PCs separately contributed 44.8% and 17.1%, respectively (Fig 1A). The scores plot revealed 2 distinct groups of metabolites associated with the drought and the control conditions between PC1 and PC2 (Fig 1B). In roots of LA754, 76.2% of the total variations were explained by the 5 PCs, with the first and second PCs contributing 44.8% and 10.9%, respectively (Fig 2A). PC1 and PC2 demonstrated 2 separate groups of metabolites that were associated with the drought and control conditions (Fig 2B). In sensitive genotype, AGS2038, 5 PCs contributed 86.0% and 74.5% of the total variations for leaf and root samples, respectively, when PLS-DA was performed across the 2 conditions and sampling times (Figs 3A and 4A). Leaf and root samples were clearly separated between drought and control conditions across the 2 sampling times in the scores plot of PC1 and PC2 (Figs 3B and 4B). This separation of samples between 2 conditions and genotypes indicate the different metabolite levels in the wheat flag leaves and roots in 2 genotypes under drought stress.

A total of 66 metabolites were identified, through a multi-factorial ANOVA, that were significantly altered either due to water treatments or across time points, or in different genotypes (S1 Table). Out of those 66, the major groups of metabolites consist of amino acid and derivatives (17), organic acids (14), sugar (11), sugar acids (5), sugar alcohol (6), fatty alcohol (5), fatty acids (3), organic compounds (2), vitamins (2), and amine (1). Two statistical methods, namely SAM and PLS-DA, were performed to identify the important metabolites associated with drought condition. A delta value of 1.4 and VIP score using the 5-component model was used to identify the most important metabolites by using SAM and PLS-DA methods, respectively. The 2 methods identified mostly the same metabolites.

**Fig 5:**
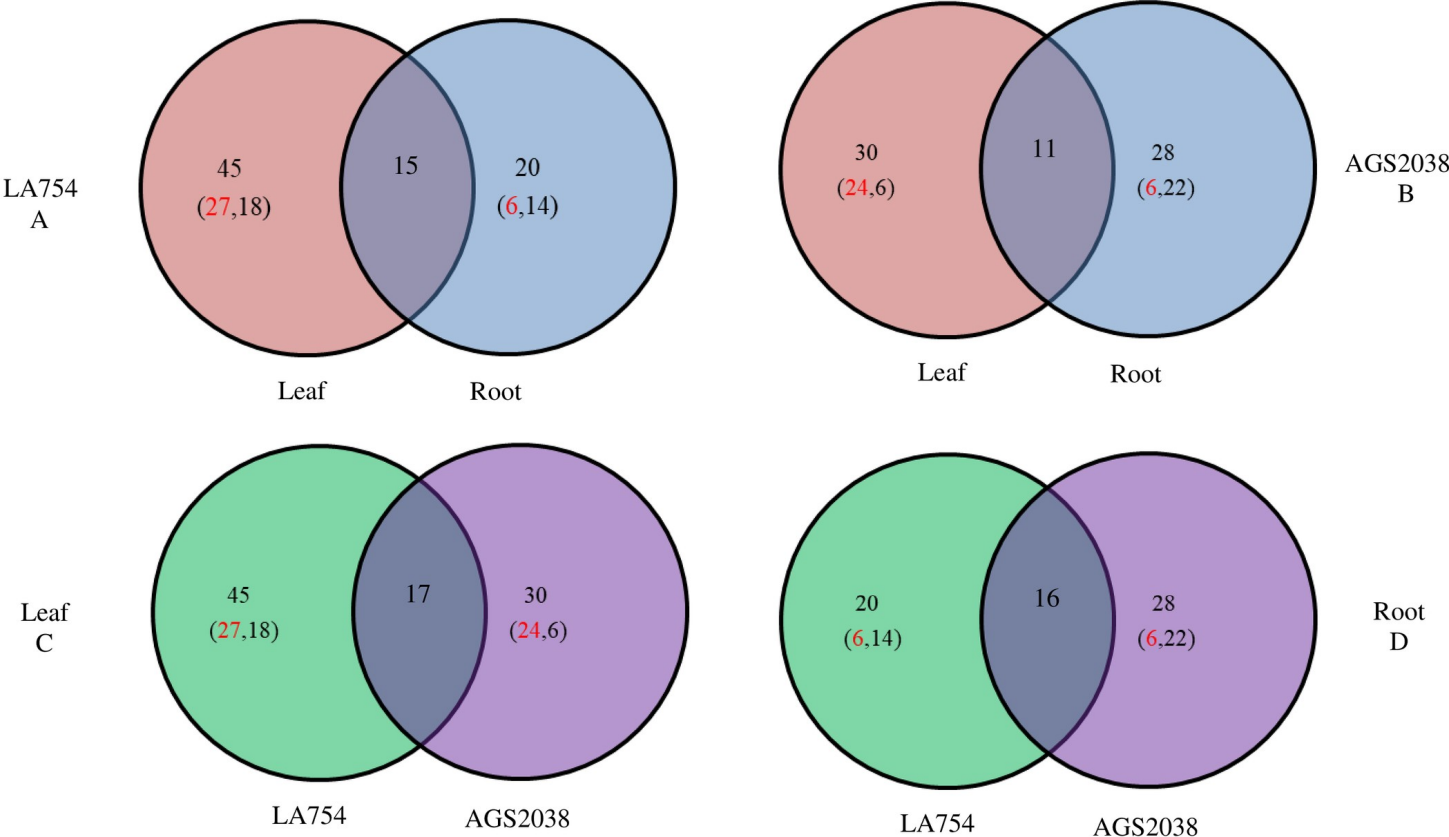
Total number of metabolites significantly regulated in drought compared to control. Colors indicate either two genotypes or leaf and root (orange = leaf, blue = root; green = ‘LA754’, purple = ‘AGS2038’). Numbers in parenthesis indicate number of metabolites up-regulated (red) and down-regulated (black).

**Fig 6:**
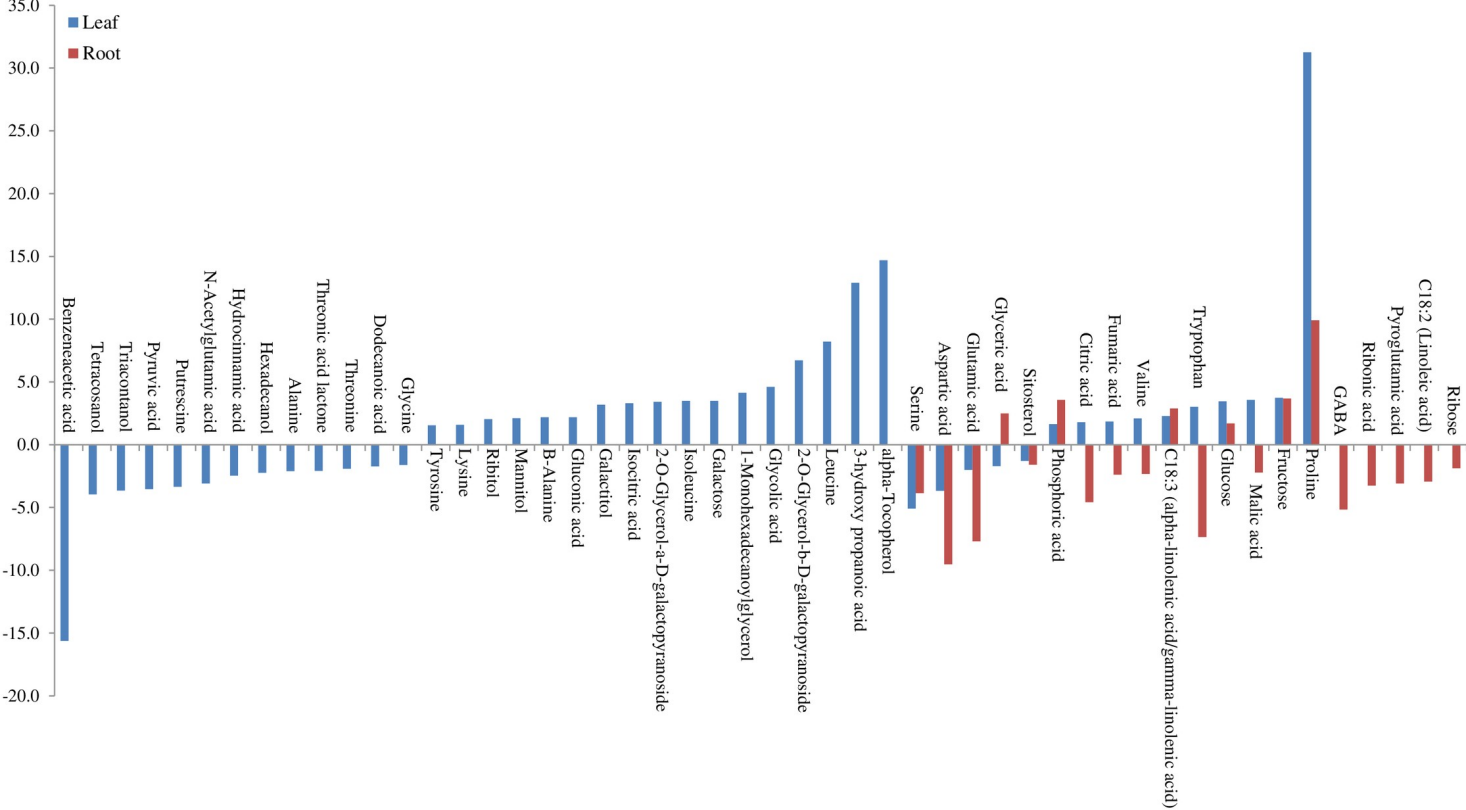
Metabolites of the absolute value of fold change (Drought/Control) which were more than 1.5 in leaf and root separately, and common in both in LA754.

In LA754, 50 metabolites were identified as most significant by SAM and PLS-DA methods (Table 3). In general, higher numbers of metabolites were altered in leaves (45) compared to roots (20) under drought stress, and 15 were commonly altered between leaf and root (Table 3 and Fig 5A). The results indicate that plants allocated more resources to leaves in tolerant genotype. Amino acids (18 in leaves, 8 in roots), organic acids (12 in leaves, 6 in roots), sugars (4 in leaves, 3 in roots), sugar alcohol (3 in leaves, 1 in roots), and fatty alcohol (3 in leaves, 1 in roots) are major groups of metabolites altered due to drought stress. In leaves, 27 metabolites were positively accumulated and 18 were decreased; however, in roots only 6 metabolites showed increased accumulation and 14 were decreased due to drought stress. Out of 15 commonly altered metabolites, proline, alpha-linolenic acid/gamma-linolenic acid, phosphoric acid, glucose, and fructose showed higher accumulation under drought condition in both leaf and root (Table 3 and Fig 6). Tryptophan, valine, citric acid, fumaric acid, and malic acid showed higher accumulation in leaf, but decreased in root (Table 3 and Fig 6). On the contrary, glyceric acid was highly accumulated in root, but not in leaf (Table 3 and Fig 6). Out of 30 metabolites, those that altered only in leaves of LA754 due to drought stress, amino acids (B- alanine, isoleucine, leucine, lysine, tyrosine), organic acids (3-Hydroxy propanoic acid, gluconic acid, glycolic acid, and isocitric acid), sugar (Galactoseand Galactitol), sugar alcohol (mannitol, ribitol, and alpha-tocopherol) showed strong accumulation due to drought stress (Table 3 and Fig 6). Contrary to leaf, all the metabolites altered only in root due to drought stress were decreased (amino acids, organic acid, fatty acid, and sugar) in their levels (Table 3 and Fig 6). In summary, a greater number of amino acids, organic acids, sugar, and sugar alcohol were accumulated higher in leaves of LA754.

**Table 3:** List of 50 important metabolites with their Kyoto Encyclopedia of Genes and Genomes identifier number identifier number (KEGG ID/PubChem CID*), molecular formula (MF), identified through Significant Analysis of Metabolites (SAM) and Partial Least Square Discrepant Analysis (PLS-DA) in LA754. T-test (P ≤ 0.05) with their p-value and fold change (FC, drought/control) in the wheat flag leaves at 14 and 21days and roots at under drought and control conditions.

	Name of metabolites	Available in Leaf (L) or Root (R)	Compound ID	Molecular Formula	Compound type	SAM (d.value)		PLS-DA (VIP score)		LA754			
										P value (t-test)		FC (drought/control)	
						Leaf	Root	Leaf	Root	Leaf	Root	Leaf	Root
1	Aspartic acid	L,R	C00049	C_4_H_7_NO_4_	Amino acid	-4.5	-6.9	1.4	1.1	0.00	0.00	-3.7	-9.5
2	Glutamic acid	L,R	C00025	C_5_H_9_NO_4_	Amino acid	-4.4	-5.4	1.4	1.1	0.00	0.00	-2.0	-7.7
3	Proline	L,R	C00148	C_5_H_9_NO_2_	Amino acid	3.0	4.2	1.2	1.0	0.00	0.00	31.2	9.9
4	Serine	L,R	C00065	C_3_H_7_NO_3_	Amino acid	-5.3	-5.1	1.4	1.1	0.00	0.00	-5.1	-3.9
5	Tryptophan	L,R	C00078	C_11_H_12_N_2_O_2_	Amino acid		-2.1	0.0	0.9	0.01	0.02	3.0	-7.4
6	Valine	L,R	C00183	C_5_H_11_NO_2_	Amino acid		-5.4	0.4	1.1	0.01	0.00	2.1	-2.3
7	C18:3 (alpha-linolenic acid/gamma-linolenic acid)	L,R	C06427/C06426	C_18_H_30_O_2_	Fatty acid		-2.8	0.4	0.9	0.00	0.00	2.3	2.9
8	Citric acid	L,R	C00158	C_6_H_8_O_7_	Organic acid		-7.9	0.4	1.1	0.00	0.00	1.8	-4.6
9	Fumaric acid	L,R	C00122	C_4_H_4_O_4_	Organic acid		-4.0	0.3	1.1	0.00	0.00	1.8	-2.4
10	Glyceric acid	L,R	C00258	C_3_H_6_O_4_	Organic acid	-4.6	3.8	1.4	0.5	0.01	0.00	-1.7	2.5
11	Malic acid	L,R	C00149	C_4_H_6_O_5_	Organic acid	2.0	-7.6	1.0	1.1	0.00	0.00	3.6	-2.2
12	Phosphoric acid	L,R	C00009	H_3_O_4_P	Organic acid		5.6	0.4	1.1	0.01	0.00	1.6	3.6
13	Fructose	L,R	C02336	C_6_H_12_O_6_	Sugar		3.0	0.7	1.0	0.01	0.00	3.7	3.7
14	Glucose	L,R	C00031	C_6_H_12_O_6_	Sugar		2.3	0.6	0.3	0.01	0.01	3.5	1.7
15	Sitosterol	L,R	C01753	C_29_H_50_O	Sugar alcohol	-3.9	-4.0	1.3	0.9	0.01	0.00	-1.3	-1.6
16	Putrescine	L	C00134	C_4_H_12_N_2_	Amine	-2.5		1.1		0.02	NS	-3.4	
17	Alanine	L	C00041	C_3_H_7_NO_2_	Amino acid	-4.0		1.4		0.01	NS	-2.1	
18	B-Alanine	L	C00099	C_3_H_7_NO_2_	Amino acid			0.4		0.01	NS	2.2	
19	Glycine	L	C00037	C_2_H_5_NO_2_	Amino acid	-4.9		1.4		0.00	NS	-1.6	
20	Isoleucine	L	C00407	C_6_H_13_NO_2_	Amino acid			0.5		0.00	NS	3.5	
21	Leucine	L	C00123	C_6_H_13_NO_2_	Amino acid	2.2		1.0		0.00	NS	8.2	
22	Lysine	L	C00047	C_6_H_14_N_2_O_2_	Amino acid	-1.4		0.8		0.02	NS	1.6	
23	N-Acetylglutamic acid	L	C00624	C_7_H_11_NO_5_	Amino acid	-3.9		1.3		0.00	NS	-3.1	
24	Threonine	L	C00188	C_4_H_9_NO_3_	Amino acid	-4.8		1.4		0.00	NS	-1.9	
25	Tyrosine	L	C00082	C_9_H_11_NO_3_	Amino acid	-1.4		0.8		0.00	NS	1.5	
26	Dodecanoic acid	L	C02679	C_12_H_24_O_2_	Fatty acid	-3.2		1.2		0.02	NS	-1.7	
27	Hexadecanol	L	C00823	C_16_H_34_O	Fatty alcohol	-3.3		1.3		0.00	NS	-2.2	
28	Tetracosanol	L	10472*	C_24_H_50_O	Fatty alcohol	-2.6		1.1		0.01	NS	-4.0	
29	Triacontanol	L	C08392	C_30_H_62_O	Fatty alcohol	-2.4		1.1		0.00	NS	-3.7	
30	3-Hydroxy propanoic acid	L	C01013	C_9_H_10_O_3_	Organic acid	3.5		1.3		0.00	NS	12.9	
31	Benzeneacetic acid	L	C07086	C_8_H_8_O_2_	Organic acid	-5.1		1.4		0.00	NS	-15.6	
32	Gluconic acid	L	C00257	C_6_H_12_O_7_	Organic acid			0.4		0.00	NS	2.2	
33	Glycolic acid	L	C00160	C_2_H_4_O_3_	Organic acid			0.5		0.02	NS	4.6	
34	Hydrocinnamic acid	L	C05629	C_9_H_10_O_2_	Organic acid	-3.9		1.3		0.00	NS	-2.5	
35	Isocitric acid	L	C00311	C_6_H_8_O_7_	Organic acid			0.4		0.00	NS	3.3	
36	Pyruvic acid	L	C00022	C_3_H_4_O_3_	Organic acid	-2.7		1.2		0.01	NS	-3.5	
37	1-Monohexadecanoylglycerol	L			Organic compound			0.6		0.02	NS	4.1	
38	2-O-Glycerol-a-D-galactopyranoside	L	46780447*	C_9_H_18_O_8_	Sugar	1.4		0.8		0.00	NS	3.4	
39	Galactose	L	C00124	C_6_H_12_O_6_	Sugar			0.5		0.01	NS	3.5	
40	Threonic acid lactone (L-Threonolactone)	L	2724794*	C_4_H_6_O_4_	Sugar acid	-3.0		1.2		0.00	NS	-2.1	
41	Galactitol	L	C01697	C_6_H_14_O_6_	Sugar alcohol			0.6		0.00	NS	3.2	
42	Mannitol	L	C00392	C_6_H_14_O_6_	Sugar alcohol			0.4		0.00	NS	2.1	
43	Ribitol	L	C00474	C_5_H_12_O_5_	Sugar alcohol	-1.7		0.9		0.00	NS	2.0	
44	alpha-Tocopherol	L	C02477	C_29_H_50_O_2_	Vitamin	-4.8		1.4		0.00	NS	14.7	
45	2-O-Glycerol-b-D-galactopyranoside	L				2.1		1.0		0.00	NS	6.7	
46	GABA	R	C00334	C_4_H_9_NO_2_	Amino acid		-6.8		1.1	NS	0.00		-5.2
47	Pyroglutamic acid	R	C01879	C_5_H_7_NO_3_	Amino acid		-5.1		1.1	NS	0.00		-3.1
48	C18:2 (Linoleic acid)	R	C01595	C_18_H_32_O_2_	Fatty acid		-4.1		1.1	NS	0.00		-2.9
49	Ribonic acid	R	C01685	C_5_H_10_O_6_	Organic acid		-5.4		1.1	NS	0.00		-3.2
50	Ribose	R	C21057	C_5_H_10_O_5_	Sugar		-2.7		0.9	NS	0.00		-1.9

Note: *PubChem ID.

**Fig 7:**
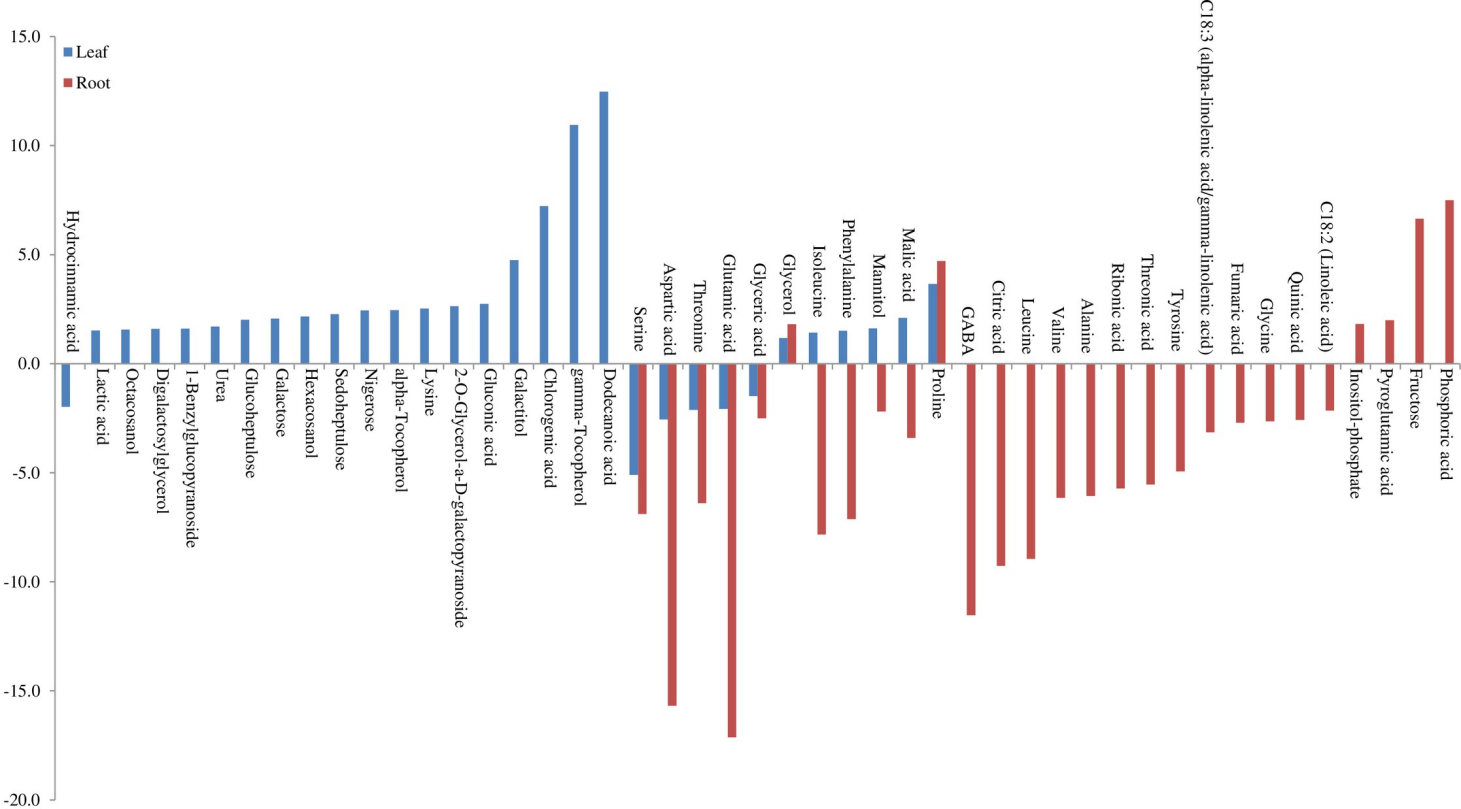
Metabolites of the absolute value of fold change (Drought/Control) which were more than 1.5 in leaf and root separately, and common in both in AGS2038.

Table 4 presents 47 important metabolites altered in AGS2038 due to drought stress identified by SAM and PLS-DA analysis, and their fold changes and t-test values. Contrary to LA754 where a significantly higher number of metabolites were altered in leaves compared to roots, and in AGS2038, a similar number of metabolites were altered in leaves and roots. Out of 47 metabolites, 30 were altered in leaves and 28 in roots, and 11 were common between roots and leaves (Figs 5B and 7). The results indicate that the plants compromised resource allocation to leaves, rather allocated more towards roots due to drought stress in sensitive genotype. Amino acids (8 in leaves, 20 in roots), organic acids (6 in leaves, 8 in roots), sugars (7 in leaves, 1 in roots), sugar alcohol (5 in leaves, 2 in roots), and fatty alcohol (1 in leaves, 2 in roots) are a major group of metabolites altered due to drought stress. In leaves, levels of 24 metabolites were increased whereas 18 were decreased; however, in roots, only 6 metabolites showed increased accumulation and 14 were decreased due to drought stress. Out of 11 commonly altered metabolites in leaf and root, proline and glycerol demonstrated higher accumulation due to drought stress in both organs, whereas amino acids (isoleucine and phenylalanine), sugar alcohol (mannitol), and organic acid (malic acid) were positively accumulated in leaf, but negatively in root. The rest of the 5 metabolites decreased their levels due to stress in both leaves and roots. Out of the 19 metabolites, those that altered their level only in leaf, fatty acid (dodecanoic acid), gamma-tocopherol, organic acid (chlorogenic acid, gluconic acid, and lactic acid), amino acid (lysine), sugars (nigerose, seduheptolose, galactose, and digalactosylglycerol), sugar alcohol (galactitol, hexacosanol, and octacosanol), and vitamins (alpha-tocopherol and gamma- tocopherol) showed strong accumulation due to drought stress (Table 4 and Fig 7). Out of the 17 metabolites in roots, those that altered levels due to drought stress, only 4 metabolites, phosphoric acid (organic acid), fructose (sugar), inositol-phosphate (organic compound), and pyroglutamic acid (amino acid) displayed higher accumulation in roots (Table 4 and Fig 7).

**Table 4:** List of 47 important metabolites with their Kyoto Encyclopedia of Genes and Genomes identifier number (KEGG ID/PubChem CID*/Metabolite ID(**)), molecular formula (MF), identified through Significant Analysis of Metabolites (SAM) and Partial Least Square Discrepant Analysis (PLS-DA) in AGS2038.

	Name of metabolites	Available in Leaf (L) or Root (R)	Compound ID	Molecular Formula	Compound type	SAM (d.value)		PLS-DA (VIP score)		AGS2038			
										P value (t-test)		FC (drought/control)	
						Leaf	Root	Leaf	Root	Leaf	Root	Leaf	Root
1	Aspartic acid	L,R	C00049	C_4_H_7_NO_4_	Amino acid	-2.092	-6.621	1.269	1.445	0.00	0.00	-2.6	-15.7
2	Glutamic acid	L,R	C00025	C_5_H_9_NO_4_	Amino acid	-1.670	-5.143	1.137	1.397	0.01	0.00	-2.1	-17.1
3	Isoleucine	L,R	C00407	C_6_H_13_NO_2_	Amino acid			0.580	0.303	0.00	0.00	1.5	-7.8
4	Phenylalanine	L,R	C00079	C_9_H_11_NO_2_	Amino acid			0.492	0.066	0.00	0.00	1.5	-7.1
5	Proline	L,R	C00148	C_5_H_9_NO_2_	Amino acid	1.416	4.560	0.903	1.363	0.01	0.02	3.7	4.7
6	Serine	L,R	C00065	C_3_H_7_NO_3_	Amino acid	-2.429		1.335	0.290	0.00	0.00	-5.1	-6.9
7	Threonine	L,R	C00188	C_4_H_9_NO_3_	Amino acid	-2.108		1.273	0.052	0.01	0.00	-2.1	-6.4
8	Glyceric acid	L,R	C00258	C_3_H_6_O_4_	Organic acid	-1.509	3.155	1.069	1.213	0.00	0.01	-1.5	-2.5
9	Malic acid	L,R	C00149	C_4_H_6_O_5_	Organic acid		1.835	0.578	0.897	0.01	0.00	2.1	-3.4
10	Glycerol	L,R	C00116	C_3_H_8_O_3_	Sugar alcohol		5.452	0.342	1.410	0.00	0.00	1.5	1.8
11	Mannitol	L,R	C00392	C_6_H_14_O_6_	Sugar alcohol		2.893	0.695	1.168	0.00	0.00	1.6	-2.2
12	Lysine	L	C00047	C_6_H_14_N_2_O_2_	Amino acid	1.400		1.009		0.00	NS	2.5	
13	Dodecanoic acid	L	C02679	C_12_H_24_O_2_	Fatty acid	-1.575		1.098		0.01	NS	12.5	
14	Chlorogenic acid	L	C00852	C_16_H_18_O_9_	Organic acid	2.323		1.317		0.00	NS	7.2	
15	Gluconic acid	L	C00257	C_6_H_12_O_7_	Organic acid	1.774		1.175		0.00	NS	2.7	
16	Hydrocinnamic acid	L	C05629	C_9_H_10_O_2_	Organic acid	-1.917		1.222		0.01	NS	-2.0	
17	Lactic acid	L	C01432	C_3_H_6_O_3_	Organic acid			0.294		0.01	NS	1.5	
18	Urea	L	C00086	CH_4_N_2_O	Organic compound			0.560		0.00	NS	1.7	
19	1-Benzylglucopyranoside	L			Sugar	1.4180		0.900		0.00	NS	1.6	
20	2-O-Glycerol-a-D-galactopyranoside	L	46780447*	C_9_H_18_O_8_	Sugar	1.550		1.088		0.00	NS	2.6	
21	Digalactosylglycerol	L	46905263*	C_15_H_28_O_13_	Sugar			0.664		0.00	NS	1.6	
22	Galactose	L	C00124	C_6_H_12_O_6_	Sugar	1.447		0.755		0.01	NS	2.1	
23	Glucoheptulose	L	ME000065**	C_7_H_14_O_7_	Sugar	1.505		1.068		0.00	NS	2.0	
24	Nigerose	L	C01518	C_12_H_22_O_11_	Sugar			0.709		0.01	NS	2.4	
25	Sedoheptulose	L	C02076	C_7_H_14_O_7_	Sugar	2.414		1.332		0.00	NS	2.3	
26	Galactitol	L	C01697	C_6_H_14_O_6_	Sugar alcohol	2.176		1.288		0.00	NS	4.7	
27	Hexacosanol	L	C08381	C_26_H_54_O	Sugar alcohol	1.991		1.243		0.00	NS	2.2	
28	Octacosanol	L	C08387	C_28_H_58_O	Sugar alcohol	1.740		1.163		0.00	NS	1.6	
29	alpha-Tocopherol	L	C02477	C_29_H_50_O_2_	Vitamin	1.427		0.965		0.00	NS	2.4	
30	gamma-Tocopherol	L	C02483	C_29_H_50_O_2_	Vitamin	1.447		1.041		0.00	NS	1.6
31	Alanine	R	C00041	C_3_H_7_NO_2_	Amino acid				0.277	NS	0.00		-6.1
32	GABA	R	C00334	C_4_H_9_NO_2_	Amino acid		-2.217		1.014	NS	0.00		-11.5
33	Glycine	R	C00037	C_2_H_5_NO_2_	Amino acid		2.588		1.106	NS	0.00		-2.6
34	Leucine	R	C00123	C_6_H_13_NO_2_	Amino acid				0.492	NS	0.01		-8.9
35	Pyroglutamic acid	R	C01879	C_5_H_7_NO_3_	Amino acid		2.764		1.143	NS	0.00		2.0
36	Tyrosine	R	C00082	C_9_H_11_NO_3_	Amino acid				0.280	NS	0.01		-4.9
37	Valine	R	C00183	C_5_H_11_NO_2_	Amino acid				0.119	NS	0.00		-6.2
38	C18:2 (Linoleic acid)	R	C01595	C_18_H_32_O_2_	Fatty acid		3.663		1.282	NS	0.00		-2.1
39	C18:3 (alpha-linolenic acid/gamma-linolenic acid)	R	C06427/C06426	C_18_H_30_O_2_	Fatty acid				0.715	NS	0.01		-3.1
40	Citric acid	R	C00158	C_6_H_8_O_7_	Organic acid		-3.444		1.255	NS	0.00		-9.3
41	Fumaric acid	R	C00122	C_4_H_4_O_4_	Organic acid		2.360		1.052	NS	0.00		-2.7
42	Phosphoric acid	R	C00009	H_3_O_4_P	Organic acid		8.095		1.466	NS	0.00		7.5
43	Quinic acid	R	C00296	C_7_H_12_O_6_	Organic acid		2.996		1.187	NS	0.00		-2.6
44	Ribonic acid	R	C01685	C_5_H_10_O_6_	Organic acid				0.251	NS	0.00		-5.7
45	Threonic acid	R	C01620	C_4_H_8_O_5_	Organic acid				0.152	NS	0.01		-5.5
46	Inositol-phosphate	R	C01177	C_6_H_13_O_9_P	Organic compound		4.655		1.369	NS	0.00		1.8
47	Fructose	R	C02336	C_6_H_12_O_6_	Sugar		5.271		1.403	NS	0.00		6.6

Note: * PubChem ID;

**NIH data repository at “UCSanDiego Metabolomics Workbench”.

**Fig 8:**
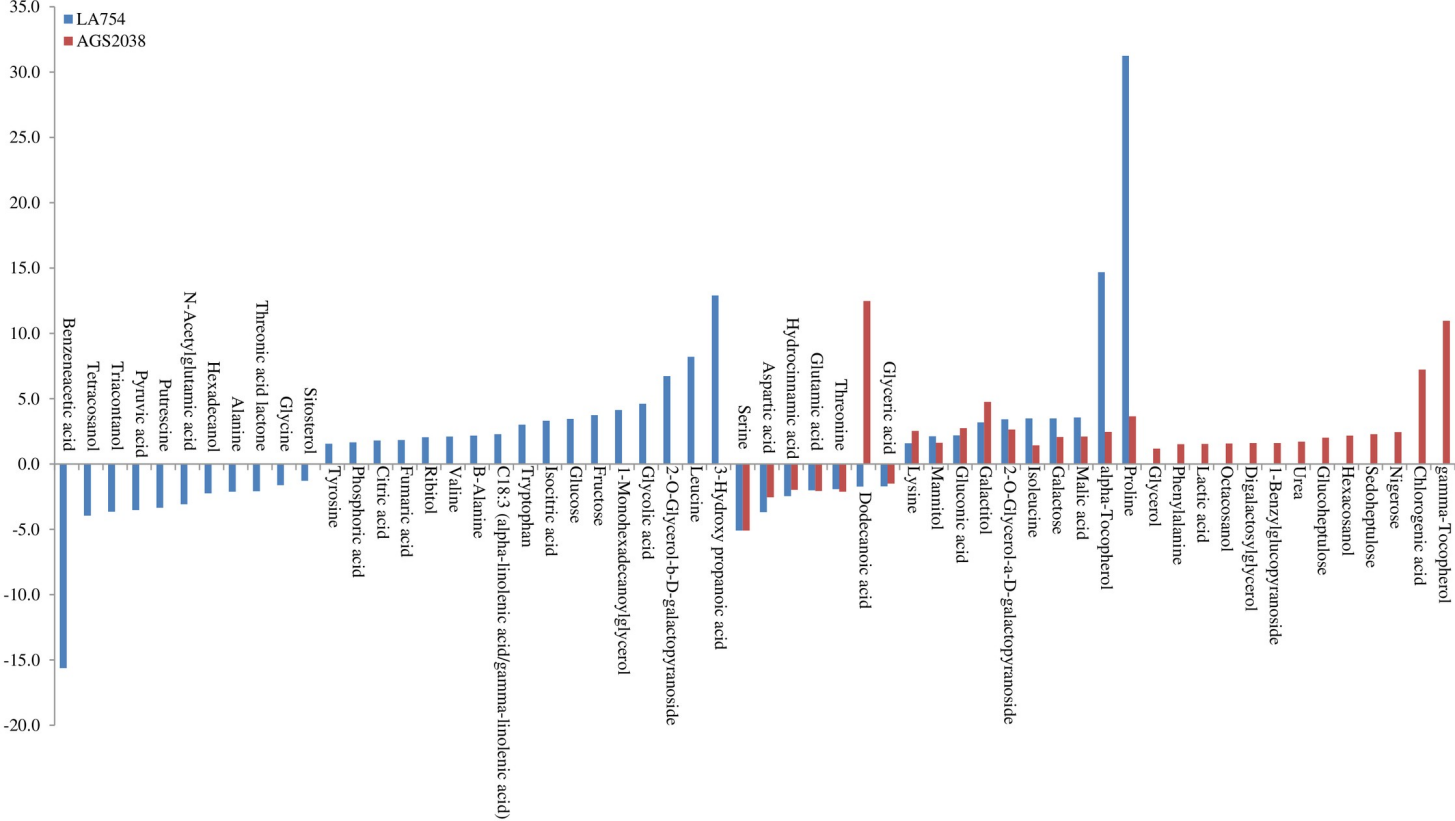
Metabolites of the absolute value of fold change (Drought/Control) were more than 1.5 in leaves.

When compared, metabolites significantly altered in leaves of 2 genotypes, 17 were common, whereas 28 were changed only in LA754 and 12 only in AGS2038 (Fig 5C). Out of the commonly altered metabolites between 2 genotypes, proline, alpha-tocopherol, malic acid, galactose, isoleucine, and mannitol were higher accumulated in LA754 compared to AGS2038, whereas lysine, galactitol, gluconic acid, and dodecanoic acid were higher accumulated more in AGS2038 than LA754 (Fig 8). Out of 28 metabolites accumulated only in LA754, amino acid (leucine, tryptophan, B-alnine, valin, and tyrosine), organic acid (3-Hydroxy propanoic acid, phosphoric acid, glycolic acid, isocitric acid, fumaric acid, and citric acid), sugar (fructose, glucose, and 2-O-Glycerol-a-D-galactopyranoside), fatty acid (alpha-linolenic acid), sugar alcohol (ribitol), and organic compound (1-Monohexadecanoylglycerol) demonstrated at least more than 1.5-fold increase under drought condition. On the contrary, in AGS2038, sugar and sugar alcohols were predominantly accumulated significantly in leaves (nigerose, seduheptulose, hexacosanol, glucoheptulose, 1-Benzylglucopyranosidem, Digalactosylglycerol, octacosanol, and glycerol). In addition, organic compounds (urea, chlorogenic acid, lactic acid), fatty acid (gamma-tocopherol), and amino acid (phenylalanine), showed a more than 1.5-fold increase under drought condition compared to well-watered condition. Comparing the 2 genotypes, leaves of LA754 accumulated predominantly amino acids, sugar, and sugar alcohol, and organic acid due to drought stress, while, in the leaves of AGS2038 under drought stress, sugar, and sugar alcohol showed higher accumulation compare to other metabolites.

**Fig 9:**
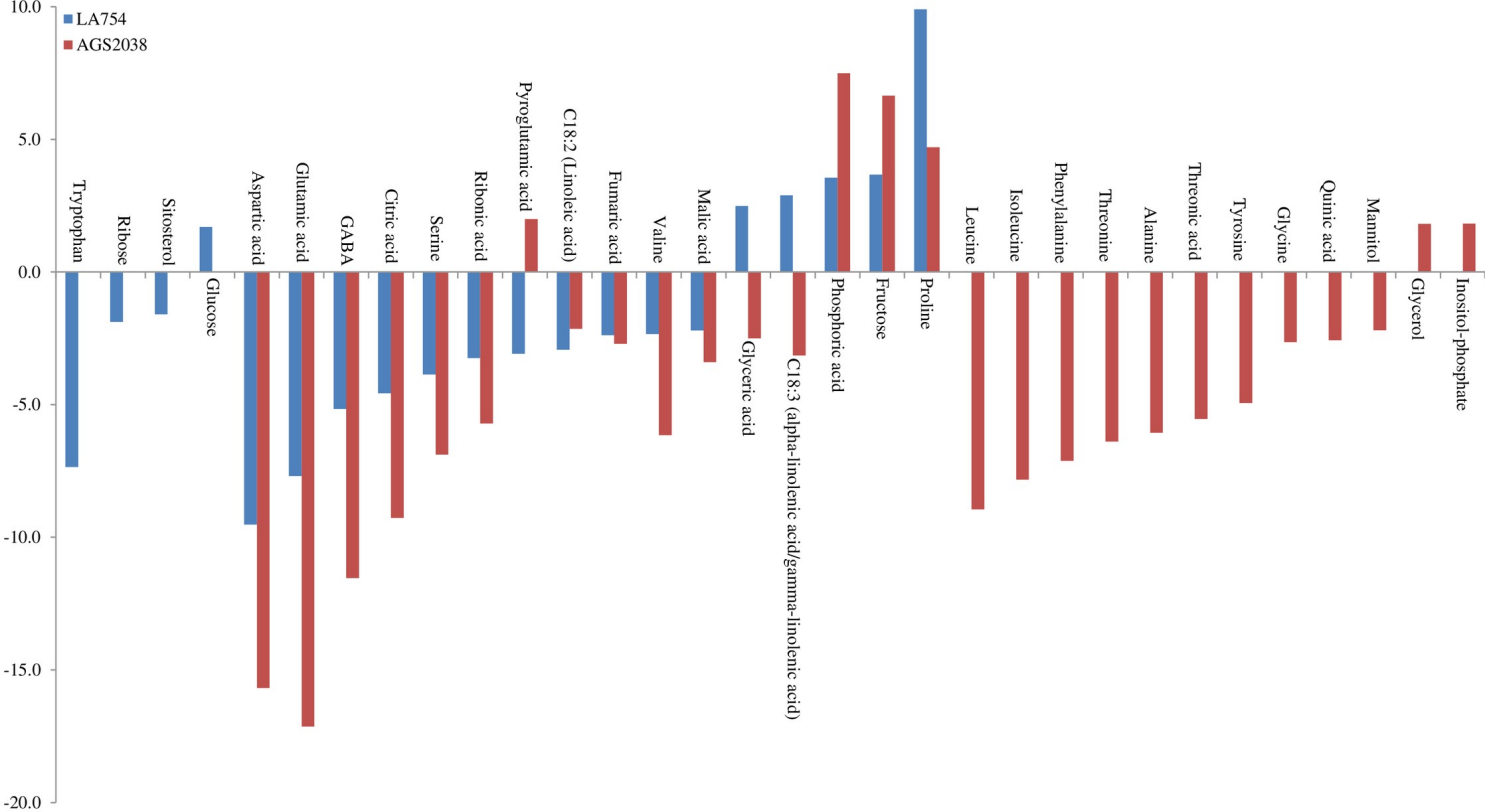
Metabolites of the absolute value of fold change (Drought/Control) were more than 1.5 in roots.

When the current study compared metabolites altered due to drought stress in roots between 2 genotypes, 16 were commonly changed, whereas only 4 were changed in LA754 and 12 were in AGS2038 (Figs 5D and 9). Out of the 16 commonly altered metabolites, proline, glyceric acid, and alpha-linolenic acid were accumulated higher in LA754 than AGS2038, whereas fructose, phosphoric acid, and pyroglutamic acid were predominantly higher accumulated in AGS2038 compared to LA754 (Fig 9). Glucose was the only metabolite that showed higher accumulation (>1.5-fold) under drought compared to the control condition in LA754, whereas glycerol and inositol- phosphate were accumulated more than 1.5-fold in AGS2038 under drought condition (Fig 9).

### Metabolic pathway analysis

The Pathway Analysis was performed on significantly altered known metabolites by using *Oriza sativa* japonica as the pathway libraries to associate the biological functions of identified metabolites to different pathways (Tables 5 and 6). In LA754, 8 different pathways were identified where different metabolites are involved in different steps (Table 5), 4 of which are common between leaves and root (aminoacyl-tRNA biosynthesis; arginine and proline metabolism; and alanine, aspartate, glutamate metabolism, and TCA cycle). Glycine, serine, and threonine metabolism; glyoxylate and dicarboxylate metabolism; and valine, leucine, and isoleucine biosynthesis pathways were significantly altered only in leaves of LA754 due to drought stress (Table 5). In AGS2038, aminoacyl-tRNA biosynthesis pathway was altered significantly by drought in both leaves and root, whereas alanine, aspartate, and glutamate metabolism, arginine and proline metabolism, and cyanoamino acid metabolism were significantly altered in root (Table 6).

**Table 5:** Pathway names, total metabolites involved in those pathways, metabolites significantly accumulated in present study (hits), and false discovery rate (FDR) in wheat flag leaves and roots of LA754 identified by Pathway Analysis of MetaboAnalyst 3 using *Oryza sativa* japonica as the pathway library.

Pathway name	Available in Leaf (L) or Root (R)	Total number of metabolites	number of metabolites were involved in		Metabolites involved in		FDR	
			Leaf	Root	Leaf	Root	Leaf	Root
Alanine, aspartate and glutamate metabolism	L,R	21	5	4	Aspartic acid, alanine, glutamic acid, fumaric acid, pyruvic acid	Aspartic acid, glutamic acid, fumaric acid, gamma-aminobutyric acid	0.010	0.017
Aminoacyl-tRNA biosynthesis	L,R	67	13	6	Glycine, aspartic acid, serine, valine, alanine, lysine, isoleucine, leucine, threonine, tryptophan, tyrosine, proline, glutamic acid	Aspartic acid, serine, valine, tryptophan, proline, glutamic acid	0.000	0.025
Arginine and proline metabolism	L,R	37	6	5	Aspartic acid, glutamic acid, N-Acetyl-L-alanine, proline, putrescine, fumaric acid	Aspartic acid, glutamic acid, proline, pumaric acid, gamma-aminobutyric acid	0.019	0.017
Citrate cycle (TCA cycle)	L,R	20	5	3	Isocitric acid, malic acid, citric acid, pyruvic acid, fumaric acid	Malic acid, Citric acid, Fumaric acid	0.010	0.027
Cyanoamino acid metabolism	L	11	3		Glycine, aspartic acid, serine		0.047	
Glycine, serine and threonine metabolism	L	29	6		Serine, glycine, aspartic acid, threonine, pyruvic acid, tryptophan		0.010	
Glyoxylate and dicarboxylate metabolism	L	17	4		Isocitric acid, glycolic acid, citric acid, malic acid		0.024	
Valine, leucine and isoleucine biosynthesis	L	26	5		Threonine, leucine, valine, isoleucine, pyruvic acid		0.020	

**Table 6:** Pathway names, total metabolites involved in those pathways, metabolites significantly accumulated in present study (hits), and false discovery rate (FDR) in wheat flag leaves and roots of AGS2038 identified by Pathway Analysis of MetaboAnalyst 3 using *Oryza sativa* japonica as the pathway library.

Pathway Name	Available in Leaf(L) or Root(R)	Total number of metabolites	number of metabolites were involved in		Metabolites involved in		FDR	
			leaf	Root	leaf	Root	leaf	Root
Aminoacyl-tRNA biosynthesis	L,R	67	8	12	Phenylalanine, aspartic acid, serine, lysine, isoleucine, threonine, proline, glutamic acid	Phenylalanine, glycine, aspartic acid, serine, valine, alanine, isoleucine, leucine, threonine, tyrosine, proline, glutamic acid	0.003	0.000
Alanine, aspartate and glutamate metabolism	R	21		5		Aspartic acid, alanine, glutamic acid, fumaric acid, gamma-aminobutyric acid		0.004
Arginine and proline metabolism	R	37		5		Aspartic acid, glutamic acidproline, fumaric acid, gamma-aminobutyric acid		0.040
Cyanoamino acid metabolism	R	11		3		Glycine, aspartic acid, serine		0.040

## Discussion

The results from the current study demonstrated significant damage (37%) to the chlorophyll content of AGS2038 (sensitive genotype) even under short-term exposure (14 days) to drought, whereas LA745 (tolerant genotype) maintained a higher chlorophyll content, and a minimal decrease was recorded both under short- and long-term drought stress. Drought stress also affected the photochemical efficiency as evident from declining value of Fv/Fm ratio, suggesting an increase in F_o_ that results inactivation of PSII. However, the percent damage was more in AGS2038 as compared to LA745. The measurement of chlorophyll fluorescence is assumed to be a valuable tool in monitoring the photosynthetic activity. The Fv/Fm ratio that is the features of the highest quantum yield of the primary photochemical reactions had been significantly reduced in drought sensitive wheat cultivars grown under water stress condition. These findings are supported by previous studies where significant decrease in leaf chlorophyll content and Fv/Fm ratio under drought stress was demonstrated [28; 33].

The improved permeability and ions leakage out of cells has been used as a measure of membrane thermostability. Membrane thermostability has been used as an idirect measurement of stress tolerance is different plant species, including wheat, as increased membrane permeability is associated higher electrolyte leakage through membrane [28]. Plasma membrane damages were associated with grain yield reduction [34] and drought tolerance [35; 36]. Similarly, drought induced significant reduction in shoot dry weight of both the tolerant and sensitive genotypes as compared to control. However, the percent reduction was less in LA754 in comparison to AGS2038. Drought induced significant decrease in the shoot dry weight of wheat is supported by others as well [37; 38].

The current study noted significant reduction in grain weight, grain number, 100-grain weight, and HI in both genotypes due to drought stress, but the percent damage was less in the tolerant genotype (LA754). In this present study, the declining photosynthetic activity and membrane integrity ultimately reflected in lower shoot biomass, grains spike^-1^, harvest index, grain weight spike^-1^, and 1000- grain weight in the sensitive genotype. The higher yield in the tolerant genotype (LA754) is characterized by a higher harvest index that was the main yield contributing trait. This was consistent with previous report that a higher harvest index is critical for higher grain yield under drought stress [39]. Drought stress might have reduced the transport and accessibility of essential nutrients in AGS2038 by rendering the root growth and proliferation as evident from poor growth and yield [23; 38].

The current study carried out GC-MS analysis to understand the metabolic alteration in different parts (root and shoot) of the plant at different growth conditions that could provide a more precise indication of stress tolerance in plants. Several previous studies linked the involvement of different defense mechanisms, including enzyme detoxification, redox balance, and signaling pathways with increased level of certain metabolites in the leaves of different plant species [40; 41; 42]. However, our knowledge is limited in understanding contrasting metabolic changes in leaves and roots in wheat and the relationship between changed metabolite levels with the performance under drought stress. The current study simultaneously analyzed the metabolic alterations in both leaves and roots that assessed the allocation of metabolites for various plant functions and avoidance of stress at the whole plant level, thereby assessing the likely contrasting responses of shoots and roots to the drought stress.

The extended level of amino acids is evident in enhancing stress pliability in plants by inducing numerous physiological mechanisms including, detoxification of ROS at photosynthetic organs, adjustment of osmotic stress, and maintenance of the intracellular pH level [43]. In this present study, enhanced accumulation of amino acids was noted due to the stress, but their relative intensity was higher in LA754 than in AGS2038. LA754 highly accumulated alanine, isoleucine, leucine, lysine, and tyrosine in leaves under drought condition, whereas AGS2038 highly accumulated isoleucine and phenylalanine. Amino acids are not only involved in the biosynthesis of proteins but also represent building blocks for numerous other biosynthetic pathways, a precursor of different secondary metabolites, and play essential roles in signaling processes in response to environmental stresses [44]. LA754 remained physiologically active for a longer duration under stress by accumulating an array of metabolites in their leaves required for better growth of plants. The enhanced level of amino acids under drought stress was reported previously in wheat [33], soybean [45], bean [46], and in chickpea [28].

Proline plays a vital role in mitigating drought stress in plants by reducing ROS level and by guarding plant cell membranes and proteins [47]. Proline was highly accumulated (88% and 53%) in the leaves and roots of LA754 under stress condition as compared to AGS2038. Accumulation of proline under stress has been associated with drought tolerance in many plants, and its concentration has been revealed to be mostly higher in tolerant genotype than in sensitive genotype [47]. It had been reported earlier that higher proline concentration in roots enhanced root hairs formation and increased root biomass thus leading to vigorous plant growth even under harsh environmental conditions [48]. Very strong accumulation of proline in the root of LA754 might contribute higher root biomass production than AGS2038 under drought condition. Proline not only acts as an osmolyte but also assists in the stabilization of cellular structure, hunting free radicals and defending cellular redox potential, improve cytoplasmic acidosis and uphold suitable NADP^+^ or NADPH ratios intimate with metabolism [28; 49].

Previous studies have shown that the enhanced level of sugar helped in evading stress by increasing the osmotic potential in plant [50].s The present study indicated an increase in sugar content with increase in drought stress. The concentration of nigrose, seduheptose, and galactose were enhanced in the leaves of AGS2038, whereas LA754 accumulated glucose, fructose, and galactose in higher concentration. Sugars play a vital role in plant growth and affect all stages of plant life cycle and interact with plant hormones and regulate growth and development in plants grown under stress [50; 51]. Sugars control cellular activity at various levels, from transcription and translation to protein stability and activity [52]. Sugars play a vital role as a signaling molecule and intricate with many metabolic processes in plants [53]. The role of glucose and fructose as a signaling molecule is well established and plays a dominant role in plant developmental processes such as plant growth, flowering, vascular tissues differentiation, and development of storage organs [54]. Sugar accumulation was higher in the roots of LA754, whereas the AGS2038 failed to accumulate sugar that might lead to a poor root system as increase in sugar concentration enhances drought resistance and acts as signal molecules [55]. The higher accumulation of amino acids and sugars in the roots of LA754 genotype might have improved the uptake of micro- and macronutrients, and thus increased root growth and plant biomass. Reduction in growth of AGS2038 may be due to the accumulation of a lesser number of drought responsive metabolites in their leaves and roots as this genotype only accumulated pyrogutamic acid in roots when exposed to stress. These results are in agreement with those reported by [56] that amino acids are linked with the growth of plant roots, symbiotic interactions, and pathogenesis in the rhizosphere.

The present study noted the accumulation of sugar alcohol, namely mannitol and oribitol in the leaves of LA754 when exposed to stress. Sugar alcohols are the prime photosynthetic products that also intricate with responses of plants to stresses and act as carbohydrate reserve in many plants under water shortage [57]. Sugar alcohols are produced externally to the chloroplast, through reductases and phosphatases, and play a vital role in osmotic stress adaptation. AGS2038 showed a positive accumulation of mannitol in their leaves but failed to accumulate in roots. Mannitol accumulation increases when plants are visible to low water potential and its accretion is controlled by inhibition of competing pathways and reduced mannitol consumption and catabolism [58; 59]. The encouraging effects of mannitol in drought and salinity tolerance in transgenic tobacco and wheat were demonstrated earlier [60; 61]. Tolerant genotype accumulated more sugar alcohol in their leaves as compared to the sensitive genotype that might lead to better growth and to enhance tolerance mechanism in LA754. Mannitol and sorbitol infiltrates through the cell wall and alters the responses of cells to low water potential [62].

The abundance of organic acids in drought tolerant genotype is induced by drought stress but the accumulation was more in leaves than in roots and in tolerant genotype than sensitive genotype. LA754 significantly accumulated 3-hydroxy propanoic acid, gluconic acid, glycolic acid, citric acid, and isocitric acid in leaves; however, fumaric acid and citric acid were negatively accumulated in their roots. In contrast, AGS2038 only accumulated gluconic acid and malic acid in leaves and phosphoric acid in roots in higher concentration. Organic acids not only act as the intermediates in energy cycle, but also play a role in plant adaptation to nutrient shortage and other abiotic stresses. Levi et al. [63] found that the accumulation of some organic acids including citric acid could contribute to greater capacity of some genotype of cotton to manage drought stress. Glyceric acid, a component of glycolysis and the TCA cycle, was significantly higher accumulated in the roots of the tolerant genotype which might have helped to maintain a healthy root system and uptake nutrients and water in the present study. The organic acids most probably played an important role as photosynthetic intermediates as evident from the metabolic expression of tolerant genotype where 50% more accumulation of organic acids occurred in the leaves as compared to the sensitive genotype. Overall, AGS2038 accumulated more organic acids (25%) in their roots as compared to LA754 under stress but could not reduce the adverse effects of stress.

Beside organic acids, fatty acids also plays an important role in different biological functions, including act as a source of reserve energy and essential components of membrane lipids in living organisms. In plants, fatty acids metabolic pathways play an important role in plant defense. The present study reported significant accumulation of alpha-linolenic acid in the leaves and roots of LA754; however, AGS2038 accumulated gamma-tocopherol only in leaves. Lenka et al. [64] reported that the upregulation of alpha-linolenic acid pathway was associated with the drought tolerance of rice genotype grown under water stress condition. They also noted that decline in the level of alpha-linolenic acid enhanced drought-induced damage in sensitive genotype. Thus, higher α-linolenic acid metabolism in plants under drought appears to be in good agreement with the inherent drought tolerance capacity in LA754 genotype. Our results clearly demonstrate that LA754 can serve as an excellent genetic resource linked with drought tolerance for genetic improvement of wheat and other cereals.

Increased level of vitamins such as alpha-tocopherol and gamma-tocopherol was noted in the current study that was also previously reported in barley [65] and chickpea [28] grown under drought stress condition. The abundant vitamins possibly act as a substitute for energy supply, assisting in carbohydrate metabolism for the production of energy and thus enhance stress tolerance in cereals [66]. LA754 significantly accumulated alpha-tocopherol in their leaves. Previous studies suggested that the most abundant form of tocopherol in leaves is alpha- tocopherol [67]. In contrast, AGS2038 accumulated both alpha-tocopherol and gamma- tocopherol; however the level of alpha-tocopherol was much lower than LA754. The roots of both the genotypes did not show any accumulation. The results from the current study are in agreement with those of Abbasi et al. [68] who reported that alpha-tocopherol is mainly accrued in the photosynthetic tissues of transgenic tobacco grown under drought stress condition but no accumulation was recorded in below ground parts. Szarka et al. [69] reported that alpha-tocopherol, ascorbate, and gluthatione form an imperative part of abiotic stress responses in plants. The concentration of alpha-tocopherol surge in plants due to ROS, and thus combat their adverse effects on plants [70].

A list of all recognized KEGG IDs was integrated into MetaboAnalyst3 and over- representation of metabolic pathway analysis was conducted. The current study demonstrated 8 different pathways in the LA754, out of which 4 were common between roots and leaves. Aminoacyl- tRNA biosynthesis pathway was commonly altered in the leaves and roots of both the genotypes (LA754 and AGS2038) under stress condition. Aminoacyl-tRNA synthetases form a different group of enzymes that certifies the transmission of genetic information from DNA into portions. This pathway contains 20 different enzymes that are responsible for esterification of tRNAs with their related amino acids and thus sustain the fidelity of protein biosynthesis process [70]. The biosynthetic pathways of some amino acids such as alanine, aspartate, and glutamate were elevated under drought condition in both the leaves and roots of LA754 and only in the roots of AGS2038 that is inconsistent with the previously published transcriptome data [71]. In the present study, the TCA cycle was upregulated in LA754 grown under water stress. The TCA cycle is an essential metabolic pathway which creates energy for different biological activities and also provides precursors used in many biosynthetic pathways [72]. During the TCA cyle, acetyle-coA, which is produced through carbohydrate, fatty acid and amino acid catabolism, is oxidize to CO_2_ to meet most of the cellular energy requirement [73]. Proline and arginine metabolism is of critical importance but not fully understood. Proline is not only a needed component of proteins but it also has a significant role to play in the adaptation to osmotic and dehydration stresses, redox control, and apoptosis. Under stress condition, the mitochondrial oxidative phosphorylation is decreased and the yield of ATP is increased through proline metabolic pathway to restore the stress induced damage [49; 74]. Similarly, arginine which is a major storage form of underground organs and roots might play a key role in nitrogen distribution and recycling in plants through its metabolic pathways [75; 76].

### Conclusion

The present study demonstrated that drought stress treated leaves and roots of wheat genotypes differing in sensitivity to drought have distinct mechanisms of metabolite accumulation and regulation that is valuable for the better understanding of overall abiotic stress tolerance mechanisms. The study demonstrated differential alteration of metabolites by drought stress, particularly in the leaves which are considered the most drought sensitive part of the plant. The metabolome of the root was not much affected by the drought stress that potentially indicates that root is more drought tolerant organ of the plant than leaves. Amino acids, organic acids, sugars, sugar alcohol, and fatty alcohol are major groups of metabolites altered due to drought stress. The metabolic composition of roots and leaves were different, and leaf metabolites were more variable than root metabolites. There is clear evidence in differences in resource allocation in two genotypes. Tolerant genotype allocated more resources in leaves than root, while sensitive genotype demonstrated similar resource allocation in roots and leaves. Some of the metabolites significantly altered only in the leaves, while some only in roots and some altered both in organs. The major metabolites those were showing significant accumulation under the drought stress were considered as the key metabolites and correlated with potential biochemical pathways, enzymes, or gene locations for a better understanding of the tolerance mechanisms. Protein synthesis cycle was active in both genotypes in both organs. However, the energy cycle was only active in both organs of tolerant genotypes (LA754). The higher accumulation of amino acids and sugars in the roots might have helped tolerant genotype to be remaining active to uptake water and nutrients, thus maintaining growth and productivity under drought stress condition. The data provided information that may, with further investigation, help to understand the biochemical pathway underlying stress tolerance in wheat genotypes.

## Supporting information

S1 Table List of 66 significantly altered metabolites (either between treatments, or genotypes, or different time points) identified through ANOVA with their p-value in roots and leaves of LA754 (tolerant genotype) and AGS2038 (sensitive genotype). NS indicates not significant.
(DOCX)

## References

[1] Xu GY, Rocha PS, Wang ML, Xu ML, Cui YC, Li LY et al (2011) A novel rice calmodulin-like gene, OsMSR2, enhances drought and salt tolerance and increases ABA sensitivity in Arabidopsis. Planta 234(1): 47–59. 10.1007/s00425-011-1386-z 21359958

[2] Obata T, Witt S, Lisec J, Palacios-Rojas N, Florez-Sarasa I, Yousfi S et al (2015) Metabolite profiles of maize leaves in drought, heat and combined stress field trials reveal the relationship between metabolism and grain yield. Plant physiol 169: 2665–2683. 10.1104/pp.15.01164 26424159 PMC4677906

[3] Lobell DB, Roberts MJ, Schlenker W, Braun N, Little BB, Rejesus RM et al (2014) Greater sensitivity to drought accompanies maize yield increase in the US Midwest. Science. 344(6183), 516–519. 10.1126/science.1251423 24786079

[4] Wang W, Vinocur B, Altman (2003) Plant response to drought, salinity and extreme temperatures towards genetic engineering for stress tolerance. Planta 218: 1–14. 10.1007/s00425-003-1105-5 14513379

[5] Kim SG, Yon F, Gaquerel E, Gulati J, Baldwin IT (2011) Tissue Specific Diurnal Rhythms of Metabolites and Their Regulation during Herbivore Attack in a Native Tobacco, Nicotiana attenuata. PLoS One 6: e26214 10.1371/journal.pone.0026214 22028833 PMC3196511

[6] Yu CQ, Huang X, Chen H, Huang G, Ni SQ, Wright JS et al (2018) Assessing the impacts of extreme agricultural droughts in China under climate and socioeconomic changes, Earth's Future 65: 689–703.

[7] Yang J, Zhang J, Wang Z, Zhu Q and Liu L (2001) Water Deficit-Induced Senescence and Its Relationship to the Remobilization of Pre-Stored Carbon in Wheat during Grain Filling. Agronomy Journal 93: 196.

[8] Zerihun A, McKenzie B & Morton J (1998) Photosynthate costs associated with the utilization of different nitrogen–forms: influence on the carbon balance of plants and shoot–root biomass partitioning. The New Phytologist 138: 1–11.

[9] Cattivelli L, Rizza F, Badeck FW, Mazzucotelli E and Mastrangelo AM (2008) Drought tolerance improvement in crop plants: An integrated view from breeding to genomics. Field Crops Research. 105: 1–14.

[10] Shi H, Chan Z (2014) Improvement of plant abiotic stress tolerance through modulation of the polyamine pathway. Journal of Integrative Plant Biology 56, 114–121. 10.1111/jipb.12128 24401132

[11] Cao Y, Luo Q, Tian Y, and Meng F (2017) Physiological and proteomic analyses of the drought stress response in Amygdalus Mira (Koehne) Yü et Lu roots. BMC Plant Biology 17: 53 10.1186/s12870-017-1000-z 28241796 PMC5327565

[12] Cornic G (2000) Drought stress inhibits photosynthesis by decreasing stomatal aperture—not by affecting ATP synthesis. Trends in Plant Science 5: 187–188.

[13] Mao W. Allington G, Li YL, Zhang TH, Zhao XY, Wang SK (2012) Life history strategy influences biomass allocation in response to limiting nutrients and water in an arid system. Polish Journal of Ecology 60: 545–557.

[14] Sims L, Pastor J, Lee T & Dewey B (2012) Nitrogen, phosphorus and light effects on growth and allocation of biomass and nutrients in wild rice. Oecologia 170: 65–76. 10.1007/s00442-012-2296-x 22407062

[15] Agren GI & Franklin O (2003) Root: shoot ratios, optimization and nitrogen productivity. Annals of Botany 92: 795–800. 10.1093/aob/mcg203 14565938 PMC4243620

[16] Carrow RN (1996) Drought Resistance Aspects of Turfgrasses in the Southeast: Root-Shoot Responses. Crop Science 36: 687–694.

[17] Lloret F, Casanovas C & Penuelas J (1999) Seedling survival of Mediterranean shrubland species in relation to root: shoot ratio, seed size and water and nitrogen use. Functional Ecology 13(2): 210–216.

[18] Poorter H & Nagel O (2000) The role of biomass allocation in the growth response of plants to different levels of light, CO_2_, nutrients and water: a quantitative review. Australian Journal of Plant Physiology 27: 595–607.

[19] Zhao Y, Zhou Y, Jiang H, Li X, Gan D, Peng X et al (2011) Systematic analysis of sequences and expression patterns of drought-responsive members of the HD-Zip gene family in maize. PloS One 6(12): e28488 10.1371/journal.pone.0028488 22164299 PMC3229603

[20] Richards RA. Reynolds MP, Rajaram S, McNab A (1996) Increasing the yield potential of wheat: manipulating sources and sinks, Increasing yield potential in wheat: breaking the barriers, Mexico, DF, CIMMYT

[21] Wen W, Li K, Alseekh S, Omranian N, Zhao L, Zhou Y et al (2015) Genetic determinants of the network of primary metabolism and their relationships to plant performance in a maize recombinant inbred line population. Plant Cell 27(7): 1839–1856. 10.1105/tpc.15.00208 26187921 PMC4531352

[22] Gargallo-Garriga A, Sardans J, Perez-Trujillo M, Rivas-Ubach A, Oravec M, Vecerova K et al (2014) Opposite metabolic responses of shoots and roots to drought. Scientific Reports 4: 6829 10.1038/srep06829 25351427 PMC4212232

[23] Ullah N, Yüce M, Neslihan Öztürk Gökçe Z, Budak H (2017) Comparative metabolite profiling of drought stress in roots and leaves of seven Triticeae species. BMC genomics 18(1): 969 10.1186/s12864-017-4321-2 29246190 PMC5731210

[24] Zhang J, Chen G, Zhao P, Zhou Q, Zhao X (2017) The abundance of certain metabolites responds to drought stress in the highly drought tolerant plant Caragana korshinskii. Acta Physiologiae Plantarum 39: 116.

[25] Kadir S, Von Weihe M, Al-Khatib K (2007) Photochemical efficiency and recovery of photosystem II in grapes after exposure to sudden and gradual heat stress. Journal of the American Society for Horticultural Science 132:764–769.

[26] Ristic Z, Bukovnik U, Prasad V (2007) Correlation between heat stability of thylakoid membranes and loss of chlorophyll in winter wheat under heat stress. Crop Science 47: 2067–2073.

[27] Ristic Z, Cass D (1993) Dehydration avoidance and damage to the plasma and thylakoid membranes in genotype of maize differing in endogenous levels of abscisic acid. Journal of Plant Physiology 142: 759–764.

[28] Khan N, Bano A, Rahman MA, Rathinasabapathi B & Babar MA (2018) UPLC-HRMS-based untargeted metabolic profiling reveals changes in chickpea (Cicer arietinum) metabolome following long-term drought stress. Plant Cell Environ. 1–18. 10.1111/pce.12956 29532945 PMC7379973

[29] Thomason K, Babar MA, Erickson JE, Mulvaney M., Beecher C., and MacDonald G. (2018) Comparative physiological and metabolomics analysis of wheat (Triticum aestivum L.) following post-anthesis at stress. PLoS ONE 13(6): e0197919 10.1371/journal.pone.0197919 29897945 PMC5999278

[30] South P, Walker B, Cavanagh A, Rolland V, Badger M, Ort D (2017) Bile Acid Sodium Symporter BASS6 Can Transport Glycolate and Is Involved in Photorespiratory Metabolism in Arabidopsis thaliana. Plant Cell. 29: 803–823. 10.1105/tpc.16.00775 PMC5435425 28351992

[31] Xia J, Mandal R, Sinelnikov IV, Broadhurst D & Wishart DS (2012) MetaboAnalyst 2.0—a comprehensive server for metabolomic data analysis. Nucleic Acids Research 40(W1): W127–W133. 22553367 10.1093/nar/gks374 PMC3394314

[32] R Development Core Team (2013) R: A Language and Environment for Statistical Computing. R Foundation for Statistical Computing, Vienna http://www.R-project.org/

[33] Rahman M, Akond M, Babar MA, Beecher C, Erickson J, Thomason K et al (2017) LC-HRMS Based Non-Targeted Metabolomic Profiling of Wheat (Triticum aestivum L.) under Post-Anthesis Drought Stress. American Journal of Plant Sciences 8:3024–3061.

[34] Blum A, Klueva N, Nguyen HT (2001) Wheat cellular thermotolerance is related to yield under heat stress. Euphytica 117: 117–123.

[35] Araus JL, Amro T, Volta J, Nakkoul H, Nachit MM (1998) Chlorophyll fluorescence as a selection criterion for grain yield in durum wheat under Mediterranean conditions. Field Crop Research 55: 209–223.

[36] Yeh DM and Lin HF (2003) Thermostability of cell membranes as a measure of heat tolerance and relationship to flowering delay in chrysanthemum. Journal of the American Society for Horticultural Science 128: 656–60.

[37] Ahemad M & Kibret M (2014) Mechanisms and applications of plant growth promoting rhizobacteria: Current perspective. Journal of King Abdulaziz University Science 26(1): 1–20.

[38] Fang Y, Du YL, Wang J, Wu AJ, Qiao S, Xu BC et al (2017) Moderate Drought Stress Affected Root Growth and Grain Yield in Old, Modern and Newly Released Cultivars of Winter Wheat. Frontiers in Plant Science 8:672 10.3389/fpls.2017.00672 28507555 PMC5410596

[39] White EM, Wilson FEA (2006) Responses of grain yield, biomass and harvest index and their rates of genetic progress to nitrogen availability in ten winter wheat varieties. Irish Journal of Agricultural and Food Research 45: 85–101.

[40] Vurukonda SSKP, Vardharajula S, Shrivastava M, SkZ A (2016) Enhancement of drought stress tolerance in crops by plant growth promoting rhizobacteria. Microbiol Research. 184 (Supplement C): 13–24. 10.1016/j.micres.2015.12.003 26856449

[41] Chen X, Chen Z, Zhao H, Zhao Y, Cheng B, Xiang Y (2014) Genome-wide analysis of soybean HD-Zip gene family and expression profiling under salinity and drought treatments. PLoS One 9: e87156 10.1371/journal.pone.0087156 24498296 PMC3911943

[42] Browne J, Erwin T, Juttner J, Schnurbusch T, Langridge P, Baci A et al (2015) Drought Responses of Leaf Tissues from Wheat Cultivars of Differing Drought Tolerance at the Metabolite Level. Mol Plant 5: 418–429. 10.1093/mp/ssr114 22207720

[43] Krasensky J & Jonak C (2012) Drought, salt, and temperature stress-induced metabolic rearrangements and regulatory networks. Journal of Experimental Botany 63(4): 1593–1608. 10.1093/jxb/err460 22291134 PMC4359903

[44] Hildebrandt TM, Nesi AN, Araújo WL & Braun HP (2015) Amino acid catabolism in plants. Molecul Plant 8(11): 1563–1579. 10.1016/j.molp.2015.09.005 26384576

[45] Silvente S, Sobolev AP & Lara M (2012) Metabolite adjustments in drought tolerant and sensitive soybean genotypes in response to water stress. PLoS One 7(6), e38554 10.1371/journal.pone.0038554 22685583 PMC3369847

[46] Sassi S, Aydi S, Hessini K, Gonzalez EM & Arrese-Igor C (2010) Long-term mannitol-induced osmotic stress leads to stomatal closure, carbohydrate accumulation and changes in leaf elasticity in *Phaseolus vulgaris* leaves. African Journal of Biotechnology, 9: 6061–6069.

[47] Hayat S, Hayat Q, Alyemeni MN, Wani AS, Pichtel J & Ahmad A (2012) Role of proline under changing environments: a review. PlantSignaling & Behavior 7(11): 1456–1466. 10.4161/psb.21949 PMC3548871 22951402

[48] Kishor PBK, Sangam S, Amrutha RN, Laxmi PS, Naidu KR, Rao KRSS et al (2005) Regulation of proline biosynthesis, degradation, uptake and transport in higher plants: Its implications in plant growth and abiotic stress tolerance. Current Science, 88: 424–438.

[49] Hare PD & Cress WA (1997) Metabolic implications of stress-induced proline accumulation in plants. Plant Growth Regulation, 21: 79–102.

[50] Wind J, Smeekens S, Hanson J (2010) Sucrose: metabolite and signaling molecule. Phytochemistry 71, 1610–1614. 10.1016/j.phytochem.2010.07.007 20696445

[51] Stokes ME, Chattopadhyay A, Wilkins O, Nambara E, Campbell MM (2013) Interplay between sucrose and folate modulates auxin signalling in Arabidopsis. Plant Physiology 162: 1552–1565. 10.1104/pp.113.215095 23690535 PMC3707552

[52] Rolland F, Baena-Gonzalez E, & Sheen J (2006) Sugar sensing and signaling in plants: conserved and novel mechanisms. Annual Review of Plant Biology 57: 675–709. 10.1146/annurev.arplant.57.032905.105441 16669778

[53] Tauzin AS, Giardina T (2014) Sucrose and invertases, a part of the plant defense response to the biotic stresses. Frontiers in plant science 23(5): 293. 10.3389/fpls.2014.00293 PMC4066202 25002866

[54] Tognetti R, Cocozza C, Marchetti M (2013) Shaping the multifunctional tree: the use of Salicaceae in environmental restoration. iForest 6: 37–47.

[55] Morkunas I, Ratajczak L (2014) The role of sugar signalling in plant defense responses against fungal pathogens. Acta Physiologiae Plantarum, 36: 1607–1619.

[56] Moe LA (2013) Amino acids in the rhizosphere: from plants to microbes. American Journal of Botany 100: 1692–1705. 10.3732/ajb.1300033 23956051

[57] Moing A (2000) Sugar alcohols as carbohydrate reserves in some higher plants. Developments in crop science 26: 337–358

[58] Pharr DM, Stoop JMH, Williamson JD, Studer Feusi ME, Conkling MA (1995) The dual role of mannitol as osmoprotectant and photoassimilate in celery. Hort Science 30: 1182–1188.

[59] Stoop JMH, Willamson JD, Conkling MA and Pharr DM (1995) Purification of NAD-dependent mannitol dehydrogenase from celery suspension cultures. Plant Physiology 108: 1219–1225. 7630943 10.1104/pp.108.3.1219 PMC157476

[60] Karakas B, Ozias-Akins P, Stushnoff C, Suefferheld M, Rieger R (1997) Salinity and drought tolerance of mannitol-accumulating transgenic tobacco. Plant, Cell & Environment 20: 609–616.

[61] Abebe T, Guenzi AC, Martin B and Cushman JC (2003) Tolerance of mannitol-accumulating transgenic wheat to water stress and salinity. Plant Physiology 131: 1748–1755. 10.1104/pp.102.003616 12692333 PMC166930

[62] Ogawa M, Yoshimori T, Suzuki T, Sagara H, Mizushima N, Sasakawa C (2005) Escape of intracellular Shigella from autophagy. Science 307: 727–731. 10.1126/science.1106036 15576571

[63] Levi A, Paterson AH, Cakmak I and Saranga Y (2011) Metabolite and mineral analyses of cotton near-isogenic genotype introgressed with QTLs for productivity and drought-related traits. Physiologia plantarum 141(3): 265–275. 10.1111/j.1399-3054.2010.01438.x 21143238

[64] Lenka SK, Katiyar A, Chinnusamy V, Bansal KC (2011) Comparative analysis of drought-responsive transcriptome in Indica rice genotypes with contrasting drought tolerance. Plant Biotechnology Journal 9(3): 315–327. 10.1111/j.1467-7652.2010.00560.x 20809928

[65] Templer SE, Ammon A, Pscheidt D, Ciobotea O, Schuy C, McCollum C et al (2017) Metabolite profiling of barley flag leaves under drought and combined heat and drought stress reveals metabolic QTLs for metabolites associated with antioxidant defense. Journal of Experimental Botany 68(7): 1697–1713. 10.1093/jxb/erx038 28338908 PMC5441916

[66] Miret JA, Munné-Bosch S (2015) Redox signaling and stress tolerance in plants: a focus on vitamin E. Annals of the New York Academy of Sciences 1340: 29–38. 10.1111/nyas.12639 25586886

[67] Grusak MA, DellaPenna D, Welch RM, (1999) Physiologic processes affecting the content and distribution of phytonutrients in plants. Nutrition Reviews. 57, S27–S33. 10568348 10.1111/j.1753-4887.1999.tb01804.x

[68] Abbasi BH, Saxena PK, Murch SJ and Liu CZ (2007). Echinacea biotechnology: challenges and opportunities. In Vitro Cellular & Developmental Biology—Plant 43: 481–492.

[69] Szarka A, Tomassovics B, Bánheghyi G (2012) The ascorbate-glutathione-(α -tocopherol triad in abiotic stress response. International Journal of Molecular Sciences 13: 4458–4483. 10.3390/ijms13044458 22605990 PMC3344226

[70] Sattler SE, Gilliland LU, Magallanes-Lundback M, Pollard M and DellaPenna D (2004) Vitamin E is essential for seed longevity, and for preventing lipid peroxidation during germination. Plant Cell 16: 1419–1432. 10.1105/tpc.021360 15155886 PMC490036

[71] Toyota K, Gavin A, Miyagawa S, Viant MR and Iguchi T (2016) Metabolomics reveals an involvement of pantothenate for male production responding to the short-day stimulus in the water flea, Daphnia pulex. Scientific reports 6: 25125 10.1038/srep25125 27113113 PMC4844948

[72] Wang D, Yin L, Wei J, Yang Z, Jiang G. (2017) ATP citrate lyase is increased in human breast cancer, depletion of which promotes apoptosis. Tumour Biol 39: 1010428317698338 10.1177/1010428317698338 28443474

[73] Desideri E, Vegliante R, and Ciriolo MR (2015) Mitochondrial dysfunctions in cancer: Genetic defects and oncogenic signaling impinging on TCA cycle activity, Cancer Letters, 356(2): 217–223. 24614286 10.1016/j.canlet.2014.02.023

[74] Hare PD, Cress WA, van Staden J (1998) Dissecting the roles of osmolyte accumulation during stress. Plant, Cell and Environment 21: 535–553.

[75] Rennenberg H, Wildhagen H, Ehlting B (2010) Nitrogen nutrition of poplar trees. Plant Biol 12:275–291. 10.1111/j.1438-8677.2009.00309.x 20398235

[76] Slocum RD (2005) Genes, enzymes and regulation of arginine biosynthesis in plants. Plant Physiology & Biochemistry 43: 729–745. 16122935 10.1016/j.plaphy.2005.06.007

