# Supplementary material for: Comparative metabolomic profiling in the roots and leaves in contrasting genotypes reveals complex mechanisms involved in post-anthesis drought tolerance in wheat
Source: PLoS One. 2019 Mar 11;14(3):e0213502. doi: 10.1371/journal.pone.0213502 (PMC6411144; doi:10.1371/journal.pone.0213502)
Supplement: S1 Table — NS indicates not significant. (DOCX) [file pone.0213502.s001.docx]

S1 Table. List of 66 significantly altered metabolites (either between treatments, or genotypes, or different time points) identified through ANOVA with their p-value in roots and leaves of LA754 (tolerant genotype) and AGS2038 (sensitive genotype). NS indicates not significant.

|  | Metabolites | Compound  type | p.value of ANOVA | | | | | |
| --- | --- | --- | --- | --- | --- | --- | --- | --- |
|  |  |  | Leaves | | | Roots | | |
|  |  |  | Condition | Genotype | Time | Condition | Genotype | Time |
| 1 | Putrescine | Amine | 0.000 | 0.001 | 0.000 | NS | NS | NS |
| 2 | Alanine | Amino acid | 0.000 | 0.000 | NS | 0.000 | 0.000 | 0.002 |
| 3 | Aspartic acid | Amino acid | 0.000 | 0.000 | 0.016 | 0.000 | 0.015 | NS |
| 4 | B-Alanine | Amino acid | 0.001 | 0.013 | 0.012 | NS | NS | NS |
| 5 | GABA | Amino acid | 0.000 | 0.000 | 0.000 | 0.000 | 0.000 | 0.011 |
| 6 | Glutamic acid | Amino acid | 0.000 | 0.000 | 0.000 | 0.000 | NS | 0.000 |
| 7 | Glycine | Amino acid | 0.000 | 0.000 | 0.000 | 0.000 | 0.001 | NS |
| 8 | Isoleucine | Amino acid | 0.014 | 0.024 | NS | 0.000 | 0.000 | 0.000 |
| 9 | Leucine | Amino acid | 0.009 | 0.024 | NS | 0.000 | 0.001 | 0.011 |
| 10 | Lysine | Amino acid | 0.000 | 0.000 | 0.000 | NS | NS | NS |
| 11 | Phenylalanine | Amino acid | 0.015 | 0.001 | NS | 0.000 | 0.001 | 0.003 |
| 12 | Proline | Amino acid | 0.000 | 0.000 | NS | NS | 0.001 | 0.000 |
| 13 | Serine | Amino acid | 0.000 | 0.000 | 0.000 | 0.000 | 0.000 | NS |
| 14 | Threonine | Amino acid | 0.000 | NS | 0.000 | 0.000 | 0.000 | 0.029 |
| 15 | Tryptophan | Amino acid | NS | NS | NS | 0.002 | 0.002 | NS |
| 16 | Tyrosine | Amino acid | 0.025 | 0.000 | NS | 0.003 | 0.007 | NS |
| 17 | Valine | Amino acid | NS | 0.028 | NS | 0.000 | 0.000 | 0.000 |
| 18 | N-Acetylglutamic acid | Amino acid derivative | 0.000 | NS | 0.001 | NS | NS | NS |
| 19 | C18:2 (Linoleic acid) | Fatty acid | NS | NS | NS | 0.000 | NS | NS |
| 20 | C18:3 (alpha-linolenic acid/gamma-linolenic acid) | Fatty acid | 0.000 | 0.000 | 0.001 | NS | NS | NS |
| 21 | Dodecanoic acid | Fatty acid | 0.025 | NS | NS | 0.014 | NS | 0.016 |
| 22 | Hexacosanol | Fatty alcohol | 0.000 | 0.001 | 0.005 | NS | NS | NS |
| 23 | Hexadecanol | Fatty alcohol | 0.004 | NS | NS | NS | NS | NS |
| 24 | Octacosanol | Fatty alcohol | 0.000 | NS | 0.014 | NS | NS | NS |
| 25 | Tetracosanol | Fatty alcohol | NS | 0.000 | 0.000 | NS | NS | NS |
| 26 | Triacontanol | Fatty alcohol | 0.000 | 0.000 | 0.001 | NS | NS | NS |
| 27 | 3-hydroxy propanoic acid | Organic acid | 0.028 | NS | NS | NS | NS | NS |
| 28 | Benzeneacetic acid | Organic acid | 0.016 | NS | NS | NS | NS | NS |
| 29 | Chlorogenic acid | Organic acid | 0.000 | 0.000 | NS | NS | NS | NS |
| 30 | Citric acid | Organic acid | 0.000 | 0.000 | NS | 0.000 | NS | 0.001 |
| 31 | Fumaric acid | Organic acid | 0.000 | 0.000 | NS | 0.000 | NS | NS |
| 32 | Glycolic acid | Organic acid | 0.000 | 0.000 | 0.000 | NS | 0.034 | NS |
| 33 | Hydrocinnamic acid | Organic acid | 0.000 | NS | NS | 0.026 | NS | NS |
| 34 | Isocitric acid | Organic acid | 0.000 | 0.000 | 0.002 | NS | NS | NS |
| 35 | Lactic acid | Organic acid | NS | NS | NS | 0.009 | 0.031 | NS |
| 36 | Malic acid | Organic acid | 0.000 | 0.000 | 0.000 | 0.000 | 0.008 | 0.000 |
| 37 | Phosphoric acid | Organic acid | 0.000 | 0.000 | 0.000 | 0.000 | 0.000 | 0.004 |
| 38 | Pyroglutamic acid | Organic acid | NS | 0.000 | NS | 0.000 | 0.009 | NS |
| 39 | Pyruvic acid | Organic acid | 0.001 | NS | NS | NS | NS | NS |
| 40 | Quinic acid | Organic acid | NS | NS | NS | 0.000 | 0.000 | NS |
| 41 | 1-Monohexadecanoylglycerol | Organic compound | 0.000 | 0.000 | 0.000 | NS | NS | NS |
| 42 | Urea | Organic compound | NS | NS | NS | 0.021 | 0.037 | NS |
| 43 | 1-Benzylglucopyranoside | Sugar | 0.001 | 0.000 | NS | NS | NS | NS |
| 44 | 2-O-Glycerol-a-D-galactopyranoside | Sugar | 0.000 | 0.000 | 0.000 | NS | NS | NS |
| 45 | 2-O-Glycerol-b-D-galactopyranoside | Sugar | NS | 0.000 | 0.000 | NS | NS | NS |
| 46 | Fructose | Sugar | 0.000 | 0.000 | 0.000 | 0.000 | 0.000 | 0.000 |
| 47 | Galactose | Sugar | 0.000 | 0.000 | 0.000 | 0.001 | 0.000 | NS |
| 48 | Glucoheptulose | Sugar | 0.000 | NS | 0.000 | NS | NS | NS |
| 49 | Glucose | Sugar | 0.000 | 0.000 | 0.000 | 0.049 | 0.000 | 0.001 |
| 50 | Inositol-phosphate | Sugar | NS | 0.000 | NS | 0.000 | 0.020 | NS |
| 51 | Nigerose | Sugar | 0.000 | 0.001 | NS | NS | NS | NS |
| 52 | Ribose | Sugar | 0.000 | 0.003 | 0.000 | 0.000 | NS | 0.023 |
| 53 | Sedoheptulose | Sugar | 0.000 | 0.000 | 0.000 | NS | NS | NS |
| 54 | Gluconic acid | Sugar acid | 0.000 | 0.000 | 0.000 | NS | NS | NS |
| 55 | Glyceric acid | Sugar acid | 0.000 | 0.005 | 0.000 | NS | NS | NS |
| 56 | Ribonic acid | Sugar acid | NS | NS | NS | 0.000 | NS | NS |
| 57 | Threonic acid | Sugar acid | 0.003 | 0.000 | NS | 0.047 | NS | NS |
| 58 | Threonic acid lactone | Sugar acid | 0.000 | 0.031 | 0.003 | NS | NS | NS |
| 59 | Digalactosylglycerol | Sugar alcohol | 0.000 | 0.010 | 0.000 | NS | NS | 0.005 |
| 60 | Galactitol | Sugar alcohol | 0.000 | NS | NS | NS | NS | NS |
| 61 | Glycerol | Sugar alcohol | 0.000 | 0.000 | 0.000 | 0.000 | NS | NS |
| 62 | Mannitol | Sugar alcohol | 0.000 | 0.000 | 0.000 | 0.000 | 0.001 | 0.034 |
| 63 | Ribitol | Sugar alcohol | 0.000 | 0.000 | 0.000 | NS | NS | NS |
| 64 | Sitosterol | Sugar alcohol | 0.000 | 0.000 | NS | 0.000 | 0.000 | 0.000 |
| 65 | alpha-Tocopherol | Vitamin | 0.000 | 0.000 | 0.000 | NS | NS | NS |
| 66 | gamma-Tocopherol | Vitamin | 0.000 | NS | 0.000 | NS | NS | NS |
